# Pharmacokinetic Parameters and Estimating Extra-Label Tissue Withdrawal Intervals Using Three Approaches and Various Matrices for Domestic Laying Chickens Following Meloxicam Administration

**DOI:** 10.3389/fvets.2022.826367

**Published:** 2022-03-03

**Authors:** Emily D. Richards, Rachel S. Dutch, Nathaniel C. Burmas, Jennifer L. Davis, Zhoumeng Lin, Maaike O. Clapham, Scott E. Wetzlich, Lisa A. Tell

**Affiliations:** ^1^Food Animal Residue Avoidance and Depletion Program, Davis, CA, United States; ^2^Department of Medicine and Epidemiology, School of Veterinary Medicine, University of California, Davis, Davis, CA, United States; ^3^Genentech, Dixon, CA, United States; ^4^Department of Biomedical Sciences and Pathobiology, Virginia-Maryland College of Veterinary Medicine, Blacksburg, VA, United States; ^5^Department of Environmental and Global Health, College of Public Health and Health Professions, University of Florida, Gainesville, FL, United States

**Keywords:** pharmacokinetics, meloxicam, domestic chicken, drug residue, meat withdrawal

## Abstract

Meloxicam is commonly prescribed for treating chickens in backyard or small commercial operations despite a paucity of scientific data establishing tissue withdrawal interval recommendations following extra-label drug use (ELDU). Historically, ELDU withdrawal intervals (WDIs) following meloxicam administration to chickens have been based on the time when meloxicam concentrations fall below detectable concentrations in plasma and egg samples. To date, no studies have addressed tissue residues. ELDU WDIs are commonly calculated using terminal elimination half-lives derived from pharmacokinetic studies. This study estimated pharmacokinetic parameters for laying hens following meloxicam administration and compared ELDU WDIs calculated using tissue terminal elimination half-lives vs. those calculated using FDA tolerance and EMA's maximum regulatory limit statistical methods, respectively. In addition, ELDU WDIs were calculated using plasma meloxicam concentrations from live birds to determine if plasma data could be used as a proxy for estimating tissue WDIs. Healthy domestic hens were administered meloxicam at 1 mg/kg intravenous (IV) once, 1 mg/kg orally (PO) once daily for eight doses or 1 mg/kg PO twice daily for 20 doses. Analytical method validation was performed and meloxicam concentrations were quantified using high-performance liquid chromatography. In general, the terminal elimination technique resulted in the longest ELDU WDIs, followed by the FDA tolerance and then EMA's maximum residue limit methods. The longest ELDU WDIs were 72, 96, and 384 (or 120 excluding fat) h for the IV, PO once daily for eight doses, and PO twice daily for 20 doses, respectively. Plasma data are a possible dataset for estimating a baseline for tissue ELDU WDI estimations when tissue data are not available for chickens treated with meloxicam. Finally, pharmacokinetic parameters were similar in laying hens to those published for other avian species.

## Introduction

Extra-label drug use (ELDU) in backyard chickens is a common practice since there are few Food and Drug Administration (FDA) approved medications for administration to individual birds or small flocks (1, 2). ELDU requires a substantially extended withdrawal interval (WDI) to avoid potentially hazardous drug residues in foods of animal origin since a regulatory approved withdrawal time (WDT) has not been established. There are several approaches for estimating an ELDU WDI. The simplest method relies on the assumption that >99% of drug is depleted after 10 elimination half-lives (3). This method commonly relies on pharmacokinetic studies focused on therapeutic use and only quantifies plasma drug concentrations. Another approach for estimating ELDU WDIs, is utilizing time vs. concentration data from classical pharmacokinetic studies and applying US or European regulatory statistical methods. These regulatory methods rely on tissue concentration data and are designed to establish a WDT for 95th or 99th percentiles of an animal population for the European Medicines Agency (EMA) or FDA, respectively (4, 5). Typical datasets used when employing these methods are expected to be normally distributed and derived from good laboratory practice studies where the animal population is homogenous and the sampling times are targeted to be above (3 sampling times) or below (2 sampling times) the tolerance (TOL) or maximum residue limit (MRL) established for human food safety. A few challenges with using these regulatory methods to estimate a WDI using published data from studies where drugs are administered ELDU include a lack of established TOLs or MRLs, sampling times focusing on therapeutic drug use rather than drug depletion, breed variation, animal subject numbers that fulfill metabolism vs. drug residue focused studies, and uneven distribution of sexes and ages of the study animals. However, because these regulatory methods use confidence interval approaches for estimating WDIs, they represent potential ranges of the population mean vs. the sample mean and could provide a more conservative ELDU WDI estimate, as required by the Animal Medicinal Drug Use Clarification Act (AMDUCA).

The elimination half-life approach is the most commonly used method by the Food Animal Residue Avoidance Databank Program (FARAD) for estimating ELDU WDIs. Ideally tissue data is preferred, however this can be challenging since many pharmacokinetic studies are focused on therapeutic use, rather than tissue residue depletion, and blood is more commonly sampled. In order to use the elimination half-life approach, there are several factors that need to be considered. Key components include confirmation that the plasma elimination half-life accurately represents the terminal elimination phase, determination if plasma concentrations reflect tissue concentrations, and the incorporation of a conservative safety factor to ensure that the ELDU WDI will apply to the majority of animals in the population being treated.

Meloxicam is a non-steroidal anti-inflammatory drug (NSAID) commonly used in avian practice (1, 6–8). Over the past 20 years, NSAIDs as a drug class have experienced drastic fluctuations in FDA-mandated labeling and usage patterns in both human and veterinary medicine (9, 10). When considering animals that produce products intended for human consumption, the Food Safety and Inspection Service (FSIS) oversees residue violations, and reported 12 animals (cattle) tested positive for violative residues of meloxicam in 2019 (11). Due to food safety concerns and the possibility that humans might consume products containing NSAID residues, FSIS developed quantitative methods for measuring multiple NSAID residues in edible tissues and the FDA has classified NSAIDs as drugs of high regulatory concern (12).

In the European Union, meloxicam is approved by the EMA for use in cats, dogs, cattle, swine and horses (13). In the United States, meloxicam is only FDA-approved for use in dogs and cats (14); however, it is commonly used extra-label in food-producing animals, including poultry. While individual companion laying hens are not as commonly used for sourcing meat for human consumption, chickens from small to mid-sized commercial operations have potential for entering the food chain (1, 15). According to AMDUCA and following regulations outlined in the US Code of Federal Regulations Title 21 part 530, licensed veterinarians with a valid veterinarian-client-patient relationship are permitted to use and prescribe FDA-approved medications in an extra-label manner (16). One stipulation of AMDUCA relating to extra-label drug use in food-producing animals is that the prescribing veterinarian must establish an extended withdrawal period for the marketing of food products based on scientific evidence and ensure that illegal drug residues do not occur (16). FARAD is a federally funded program that serves to help US veterinarians by recommending scientifically-based WDIs following ELDU. According to FARAD internal WDI request data, meloxicam was the most-requested drug in poultry between 2015 and 2020 (15). Requests during this time period were for meloxicam administered parenterally (intravenous, intramuscular or subcutaneous) and orally at doses ranging from 0.2 mg/kg once to up to 3 mg/kg twice daily for long-term administration. However, the most commonly requested dose for WDI submissions was 1 mg/kg, therefore this study was completed at 1 mg/kg using various routes: IV to obtain maximum concentrations for comparison between plasma and tissue samples and accurately determine a volume of distribution, and multiple PO doses to more closely replicate clinically relevant dosing regimens.

Plasma pharmacokinetic parameters for meloxicam have been extensively described in the literature for avian species, including domestic broiler chickens (8, 17–21). However, chicken studies evaluating meloxicam residues have focused only on eggs. Furthermore, no pharmacokinetic studies have compared drug concentrations in plasma samples from live animals to plasma or tissue samples collected at slaughter, despite the necessity of quantifying or comparing drug concentrations in these different matrices in order to accurately predict WDIs following ELDU.

The purpose of this study was to use plasma and tissue concentration vs. time data from classical pharmacokinetic studies following administration of various dosing regimens of meloxicam to chickens to (1) compare estimated ELDU WDIs calculated using terminal elimination half-lives with ELDU WDIs calculated using FDA and EMA statistical methods; (2) determine the relationship between plasma drug concentrations and tissue drug concentrations for multiple tissues; (3) estimate non-compartmental pharmacokinetic parameters for laying hens (*Gallus gallus domesticus*).

## Materials and Methods

### Animals

#### Intravenous Administration

Twenty-five purpose-bred, adult (~21 months old) commercial laying hens (W-36, Hy-line, Des Moines, IA, USA) were initially enrolled. Chicken body weights ranged from 1.6 to 2.3 kg (mean ± SD of 1.89 ± 0.18 kg) and birds were considered healthy based on physical examination.

#### Oral Administration

**Figure 1:**
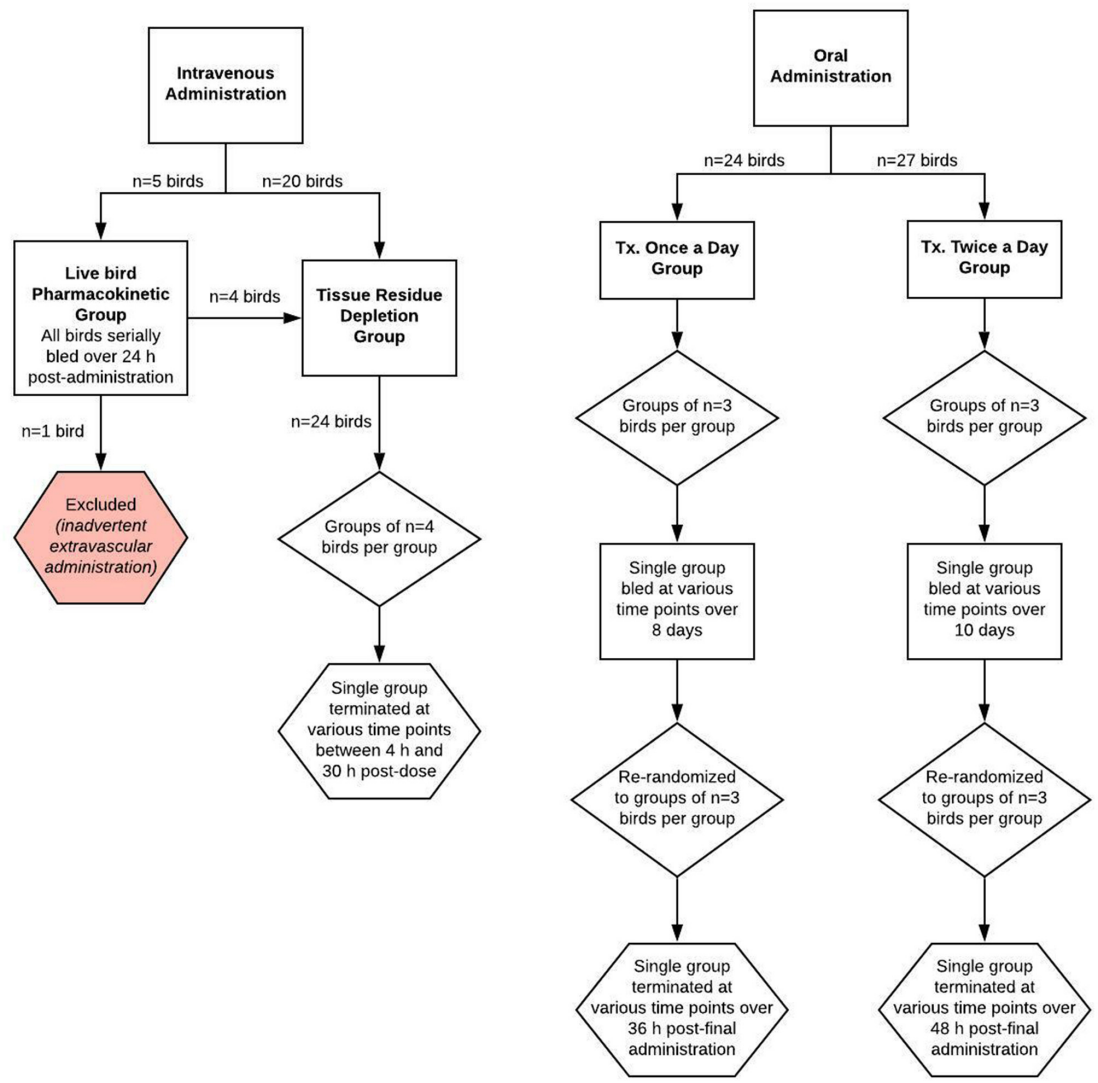
Schematic of the study design for evaluating meloxicam depletion in plasma and tissue samples from adult female laying chickens following once or twice daily oral or intravenous administration of meloxicam at a dosage of 1 mg/kg body weight.

Fifty-one purpose-bred commercial laying hens (W-36, Hy-line, Des Moines, IA, USA) were initially enrolled in two dosing regimen groups (once daily administration or twice daily administration; Figure 1). Ages ranged from 8 to 19 months and birds were classified as adult animals based on their laying status and industry standards (22). Chicken body weights ranged from 1.4 to 2.2 kg (mean ± SD of 1.9 ± 0.17 kg) for the once daily group and from 1.2 to 1.6 kg (mean ± SD of 1.42 ± 0.102 kg) for the twice daily group, and birds were considered healthy based on physical examination.

Throughout the duration of the study, chickens were housed at the University of California, Davis Hopkins Avian Facility in wire cages with the ability to see other chickens. Birds were kept in a climate-controlled room with a 16 h light and 8 h dark cycle. Chickens were fed a commercial poultry feed (16% Layer Crumble Pak, Bar ALE, Williams, CA, USA) and provided water *ad libitum*, as well as supplemented with oyster shell and a ground performance-enhancing supplement (Calf Manna Pro, Chesterfield, MO, USA). Birds were observed once daily by facility personnel evaluating physical health and fecal matter consistency, as well as to collect any eggs. Birds were physically inspected for complications relating to experimental procedures each time they were handled by investigators. Procedures relating to this study were performed in accordance with a protocol approved by the Institutional Animal Care and Use Committee of the University of California, Davis (IACUC Protocol Number 21269).

#### Organ Weights

A total of 10 purpose-bred, adult (ranging from 8 to 21 months old) commercial laying hens (W-36, Hy-line, Des Moines, IA, USA) had their organ weights quantified during necropsy as part of the tissue sample analysis. Five of these birds participated in the IV dosing regimen group while the remaining five birds were untreated. Chicken body weights ranged from 1.6 to 2.1 kg (mean ± standard deviation of 1.81 ± 0.19 kg). Specific organ weight data can be found in Supplementary Table 1.

### Experimental Protocol

#### Intravenous Administration

A total of 25 birds were used for two investigations: a live bird pharmacokinetic portion, where the same birds were sampled for the duration of the investigation, and a drug residue depletion portion, where both plasma and tissue matrices were harvested. Four of the birds were used for both investigations. Hens were administered a single intravenous bolus dose (1 mg/kg) of meloxicam (meloxicam 5 mg/ml solution for injection, Dechra, Overland Park, KS, USA) using 1 mL tuberculin syringes. Birds were weighed prior to meloxicam administration in order to provide an accurate individualized dose and weighed again at euthanasia. Five of the hens were administered meloxicam via direct syringe and needle venipuncture of the cutaneous ulnar vein, while the remaining 20 birds were administered meloxicam via a 25G × $\frac{3}{4}$″ winged infusion set in the cutaneous ulnar vein. Choice of administration method was based on wing conformation.

For the live bird pharmacokinetic investigation, five randomly selected birds were serially bled at 5 min, 15 min, 30 min, 1, 2, 3, 4, 6, 8, 12, 18, and 24 h post-dose. In accordance with IACUC blood volumes allowed for sampling, ~0.5–1 mL of blood was drawn from each bird at each time point via needlestick from one of the following veins: right or left basilar vein (medial wing vein) or medial metatarsal vein; then transferred into a 2 mL sodium heparin blood tube. Blood tubes were placed on ice, centrifuged at 2,730 × g for 10 min at 21°C, then plasma samples were manually harvested and transferred to storage tubes. Storage tubes were immediately frozen at −20°C for up to 36 h until transport to the analytical laboratory, after which they were stored at −70°C until analysis. After blood was drawn at the 24 h post-dosing time point, four of the five birds (one bird was excluded due to extravascular drug administration) were sacrificed according to the process described below for the tissue residue depletion investigation.

For the ELDU tissue drug depletion investigation, 24 birds (of which 4 were from the live bird pharmacokinetic portion) were divided into six equal groups, which fulfilled FDA requirements for numbers of birds required for a drug metabolism study (23) and time points required for establishing a withdrawal period determination. A single group (*n* = 4 birds/group) was sacrificed via CO_2_ asphyxiation at 4, 8, 12, 18, 24, or 30 h post-dose. Immediately following death, blood and tissues were harvested from each hen (2 mL of blood via cardiac stick, the entire liver, both kidneys; 10-g specimens of both breast muscles, thigh muscle and adipose) and stored frozen at −70°C until analysis.

#### Oral Administration

Two investigations were completed using different dosing regimens of oral meloxicam: 1 mg/kg once daily for eight doses and 1 mg/kg twice daily for 20 doses.

For the once daily dosing regimen group, a total of 24 birds were randomly divided into groups of three and administered meloxicam suspension (Meloxidyl 1.5 mg/mL, Ceva Animal Health, Lenexa, KS, USA) using 1 mL tuberculin syringes. Dosing syringes were gently placed into the opening of the esophagus slightly to the right and caudal of the glottis. The dosing syringe plunger was pushed slowly to dispense the suspension and the bird was visually monitored for regurgitation. Birds were weighed prior to meloxicam administration in order to provide an accurate individualized dose and weighed again at euthanasia to assess if any weight change occurred. Blood was collected from all birds at baseline (*t* = 0) and from a single group at 8.75, 39.5, 54.7, 74.5, 108.25, 120.75, 148, and 188.5 h after the first dose. After the final administration, birds were sacrificed in groups (*n* = 3) at 1, 4, 6, 8, 12, 16, 24, and 36 h after the final dose.

For the twice daily dosing regimen group, a total of 27 birds were randomly divided into groups of three and administered meloxicam and weighed as for the once daily dosing regimen group. Blood was collected from all birds at baseline (*t* = 0) and from a single group at 12.5, 24.75, 36.5, 49, 66, 83.5, 86, 100, 115, 129, 142, 146, 158.5, 176, 182, 203, 204, and 219 h after the first dose. After the final administration, birds were sacrificed in groups (*n* = 3) at 1, 4, 6, 8, 12, 16, 24, 36, and 48 h after the final dose. For both oral dosing regimen groups, blood and tissues were collected and handled in the same manner as described in the intravenous tissue drug depletion investigation above.

### Chemicals and Reagents

The analytical grade meloxicam was a European Pharmacopeia reference standard. Piroxicam as the internal standard was purchased from Alfa Aesar (Ward Hill, MA, USA). HPLC-grade methanol and acetonitrile, dimethyl sulfoxide, potassium phosphate monobasic, phosphoric acid and sodium sulfate were purchased from Fisher Scientific (Fair Lawn, NJ, USA). Purified water was obtained with a Nanopure water system (Barnstead, Dubuque, IA, USA).

### Analytical Methods

Chromatographic conditions, preparation of standards and quality control samples, and tissue sample cleanup were adapted from Depenbrock et al. (24).

The HPLC system consisted of an Alliance 2695 separations module and a 2996 photodiode array detector (Waters, Milford, MA, USA). Separation was achieved on a Nova-Pak C18, 4-μm, 300 × 3.9 mm column (Waters, Milford, MA, USA). The column temperature was maintained at 30°C and the samples were kept at 10°C. The isocratic mobile phase was a 50:50 mixture of 50 mM potassium phosphate buffer (pH 2.15) and acetonitrile set at a flow rate of 0.8 mL/min. Injection volume was 50 μL. Peaks were detected at a wavelength of 355 nm and the total run time was 10 min.

*Preparation of standards and quality control samples:* A primary stock solution of meloxicam (1.0 mg/mL) was prepared in dimethyl sulfoxide and diluted to a secondary stock solution (0.1 mg/ml) in 50% methanol. The secondary stock solution was used to create a series of working standard solutions (40–20,000 ng/mL), also in 50% methanol, and were prepared fresh for each analysis. A primary stock solution of piroxicam (1.0 mg/mL) in dimethyl sulfoxide and a secondary stock solution (0.1 mg/mL) in 50% methanol were similarly prepared. A 500 ng/mL working solution in 50% methanol was diluted from the secondary stock solution. Equal volumes of the meloxicam working solutions and the internal standard working solution were mixed for the standard curve (20–2,500 ng/mL or 16–400 ng/mL, plasma or tissue). Three different concentrations of quality control samples (20, 100, and 400 ng/g or ng/mL) were prepared in control matrix with each analysis along with a matrix blank. Control matrices were collected from liver, kidney, thigh muscle, breast muscle and adipose from non-medicated hens at the time of slaughter. Control plasma was harvested from non-medicated hens via cardiac stick immediately following slaughter; additional control plasma was obtained via a commercial source (Innovative Research, Novi, MI, USA).

*Sample preparation:* Tissue samples were weighed and processed with a commercial food processor (Little Pro Plus, Model LPP, Conair, Stamford, CT, USA). Duplicate 1 g aliquots were weighed into centrifuge tubes and spiked with 200 μL of the working internal standard solution. Samples were then extracted with 20 mL of acetonitrile and 10 mL of hexane on a platform shaker for 10 min at 250 rpm. Tissue samples were homogenized with a Polytron between addition of the acetonitrile and hexane before being placed on the shaker. After centrifugation at 1,200 × g for 10 min the hexane was removed to waste and the acetonitrile was transferred to a 25 mL volumetric flask. Samples were brought to volume, added to 15 g of sodium sulfate, shaken for 1 min and centrifuged again at 1,200 × g for 10 min. A 12.5 mL aliquot of the extractant was evaporated to dryness at 50°C with a gentle stream of nitrogen, reconstituted with 200 μL of 50% methanol and centrifuged at 12,000 × g for 5 min before analysis on the HPLC system.

Single plasma samples (250 μL) were spiked with 50 μL of the working internal standard solution prior to the addition of 3.5 mL of acetonitrile. After vortex mixing, samples were centrifuged at 1,200 × g for 10 min. The extractant was transferred to a new tube and evaporated to dryness at 50°C with a gentle stream of nitrogen, reconstituted with 100 μL of 50% methanol and centrifuged at 12,000 × g for 5 min before analysis on the HPLC system.

### Method Validation

Plasma and tissue methods were validated according to the FDA Bioanalytical Method Validation Guidance for Industry (25). Representative calibration plots and chromatograms for analysis of meloxicam in chicken plasma and tissues (liver, kidney, breast muscle, thigh muscle and adipose) are available in Supplementary Figures 1–7. Intra-day precision was calculated on a single day using five replicates at each concentration and inter-day precision was calculated using five replicates at each concentration over three consecutive days. Calibration curves were created using the ratio of meloxicam to the internal standard peak areas and had a 1/(X^*^X) weighting. The average R squared was 0.9963. Limit of detection (LOD) was calculated by adding three times the standard deviation of baseline measurements to the average baseline measurement using the blank quality controls analyzed with each sample set. Following the FDA Guidance for Industry, the lower limit of quantification (LLOQ) was measured as five times the baseline measurement. The three quality control levels described above under, “*Preparation of standards and quality control samples*” were also used to measure precision and accuracy of the method concurrent with sample analysis. Table 1 includes plasma and tissue LLOQ/LODs for meloxicam, as well as the average precision and accuracy using relative standard deviation.

**Table 1:** Sensitivity, precision, and accuracy parameters for the high-performance liquid chromatography analytical method used to measure meloxicam concentrations in various matrices from chickens following meloxicam dose administered to laying hens.

**Matrix**	**LOD (ng/mL)**	**LLOQ (ng/mL)**	**Precision (%)**	**Accuracy (%)**	**Intra-assay variation (%)**
Plasma	4.3	15	5.0	98.5	4.4
Breast muscle	4.1	10	3.5	105.2	3.1
Adipose	4.1	10	5.2	97.8	4.2
Kidney	3.6	15	4.4	100.4	3.4
Liver	5.2	15	4.0	97.2	4.0
Thigh muscle	3.0	6	4.8	104.0	4.1

### Pharmacokinetic Analysis

Concentration vs. time data for plasma and tissues from each dosing regimen group were used to estimate plasma pharmacokinetic parameters using a commercial software program (Phoenix WinNonLin 8.1, Certara, Princeton, NJ, USA) and a non-compartmental analysis approach. Terminal elimination-half lives were estimated using the best fit data points. For tissue samples, terminal elimination half-lives were estimated using a naïve-pooled data approach. The pharmacokinetic parameters were calculated as follows: area under the plasma concentration-time curve extrapolated to infinity (AUC_0−∞_) using the linear trapezoidal method, elimination rate constant (λ_z_) using a linear regression of the terminal log-linear portion of the plasma or tissue concentration profile, terminal elimination half-life using the quotient of dividing the natural log of 2 by the elimination rate constant, volume of distribution (V_d_) using the product of clearance multiplied by mean residence time, clearance (CL) using the quotient of dividing the dose by the area under the plasma concentration-time curve, and mean residence time extrapolated to infinity (MRT_0−∞_) using the quotient of dividing the area under the moment curve by the area under the plasma concentration-time curve. Concentration vs. time data for the plasma and tissue samples collected were plotted using a commercial graphing software (GraphPad Prism 9.0.0, GraphPad Software, La Jolla, CA, USA).

### Calculation of Estimated Extra-Label Drug Use Withdrawal Intervals

Terminal elimination half-lives were used to estimate ELDU WDIs relative to the time when >99% of the drug would be expected to be eliminated from the body (26). For the IV live bird pharmacokinetic investigation, this preliminary WDI estimate was based on the mean (*n* = 4 birds) plasma sample terminal elimination half-life multiplied by 10. A minimum of 5 time points from the elimination phase of the plasma concentration vs. time curve were included in the estimate of the elimination half-life. Similarly, plasma and tissue terminal elimination half-lives derived using a naïve-pooled approach from the IV residue depletion portion and PO dosing regimen groups were used to estimate WDIs.

Concentration vs. time data for plasma and tissues collected from each bird slaughtered were used to estimate WDIs according to the FDA and EMA guidance using open source statistical programs (FDA “reschem” R package, EMA Withdrawal-Time Calculation-Program WT 1.4) (4, 5). To assess linearity, linear models of the logarithmic concentration vs. time curve were run for each permutation of inclusion of time points. The model with the lowest *p*-value was considered the best linear model, and any time points not included in this chosen model were excluded. Additionally, any time point where a majority or all concentration values were below LLOQ was dropped from analysis. Since meloxicam is not approved in the US or EU for poultry, there is no established TOL or MRL. Following the FDA regulatory guidance, the LOD for plasma and each tissue was applied in place of the TOL and any data points below the LOD were excluded from calculations in ELDU WDI estimations using the FDA tolerance method (5). For ELDU WDI estimations using the EMA MRL method, the following EMA regulatory guidelines were used: (1) LLOQ was doubled then used in place of an MRL and (2) any concentration data point greater than LOD but less than LLOQ was converted to LLOQ divided by 2 prior to analysis (4, 27).

Once the raw WDI estimates were obtained for each matrix, the overall recommended slaughter withhold was determined by rounding the edible tissue matrix with the longest WDI estimate up to the nearest 24 h interval.

## Results

Throughout the entire study period, birds remained in good health without any visible side effects following meloxicam administration. Eggs and reproductive tissues collected were analyzed for meloxicam concentrations and results are reported in a companion manuscript (28). For the IV live bird plasma pharmacokinetic investigation, a single bird was excluded due to inadvertent extravascular meloxicam administration, therefore data is available from only four birds and the WDI is labeled as preliminary. In the PO twice daily dosing regimen group, samples were excluded from one bird that died prior to study completion. Subsequent necropsy and histologic examination determined the bird died due to hemorrhagic liver syndrome, which was considered to be unrelated to drug administration.

**Figure 2:**
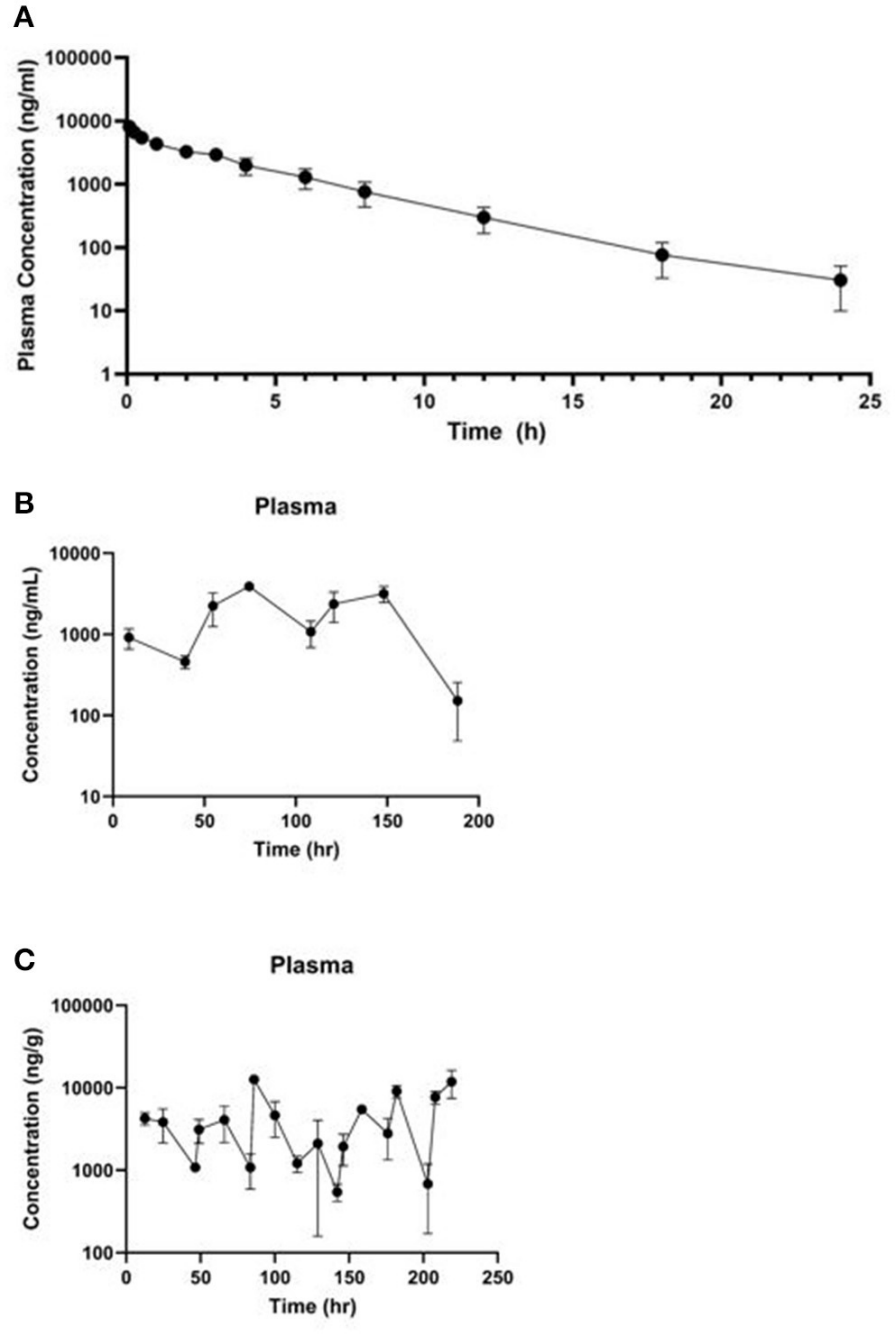
Mean plasma ± standard deviation concentration vs. time curve collected **(A)** over 24 h from four live animals following a single 1 mg/kg IV dose of meloxicam in chickens, **(B)** 188.5 h after the first dose of a 1 mg/kg PO once daily for 8 dose regimen, and **(C)** 219 h after the first dose of a 1 mg/kg PO twice daily for 20 dose regimen.

The mean meloxicam plasma concentration vs. time profile for the three dosing regimens are presented in Figure 2 (IV live birds in A, PO once daily in B, PO twice daily in C) and the associated plasma pharmacokinetic parameters are provided in Table 2 (IV) and Table 3 (PO). Overall, the plasma concentrations collected from the IV live birds were comparable to plasma concentrations from cardiac puncture samples collected at slaughter for the same post-dosing time points (Supplementary Table 2). Tissue pharmacokinetic parameters for the IV portion are presented in Table 4. Given the necessary later times for tissue sampling following oral administration, limited pharmacokinetic parameters are reported.

**Table 2:** Pharmacokinetic parameters estimated from meloxicam concentrations in plasma samples collected from live birds (pre-slaughter) following a single 1 mg/kg IV dose administered to laying hens (*n* = 4).

**Parameter**	**Geometric mean (Range)**
C_0_ (μg/mL)	8.92 (7.57–10.06)
T_max (obs)_ (h)	0.083
Terminal elimination half-life (h)	3.08 (2.75–3.58)
λ_z_ (1/h)	0.224 (0.194–0.252)
V_d_ (mL/kg)	190.1 (145.9–226.6)
CL (mL/min/kg)	0.71 (0.565–0.951)
AUC_0−∞_ (h*μg/mL)	23.45 (17.53–29.52)
AUC extrapolation (%)	0.61 (0.39–1.1)
MRT_0−∞_ (h)	3.88 (3.12–4.66)

*C_0_, extrapolated plasma concentration at time = 0; T_max (obs)_, observed time to maximum plasma concentration; λ_z_, terminal elimination rate constant; V_d_, volume of distribution; CL, clearance; AUC_0−∞_, area under the plasma concentration time curve from time 0 to infinity; MRT_0−∞_, mean residence time. The terminal elimination half-life was calculated based on the best fit data points*.

**Table 3:** Pharmacokinetic parameters estimated from meloxicam concentrations in plasma samples collected from live birds (pre-slaughter) and at slaughter following two oral meloxicam dosing regimens (1 mg/kg once daily for 8 doses or 1 mg/kg twice daily for 20 doses) administered to laying hens.

**Parameter**	**Once daily dosing regimen**		**Twice daily dosing regimen**	
	**After 1st dose**	**After last dose**	**After 1st dose**	**After last dose**
C_max (obs)_ (μg/mL)	3.896	3.54	11.86	6.69
T_max (obs)_ (h)	2.5	1	3	1
Terminal elimination half-life (h)	2.94	3.49	2.54	4.74
λ_z_ (1/h)	0.236	0.199	0.273	0.146
V_d_/F (mL/kg)	142	168.33	76.13	199.78
CL/F (mL/min/kg)	0.559	0.557	0.347	0.487
AUC_0−∞_ (h*μg/mL)	29.84	29.91	48.05	34.21
AUC extrapolation (%)	2.15	0.18	8.22	1.02
MRT_0−∞_ (h)	6.65	7.05	5.13	6.32

*C_max (obs)_, maximum observed plasma concentration; T_max (obs)_, observed time to maximum plasma concentration; λ_z_, terminal elimination rate constant; V_d_/F, apparent volume of distribution; CL/F, apparent clearance; AUC_0−∞_, area under the plasma concentration time curve from time 0 to infinity; MRT_0−∞_, mean residence time. The terminal elimination half-life was calculated based on the best fit data points*.

**Table 4:** Tissue pharmacokinetic parameters estimated from meloxicam concentrations in samples collected from birds at slaughter (at 4, 8, 12, 18, 24, and 30 h) following a single 1 mg/kg intravenous meloxicam dose administered to laying hens.

**Parameter**	**Breast muscle**	**Kidney**	**Liver**	**Thigh muscle**	**Adipose**
C_max (obs)_ (μg/mL)	0.0684	0.797	0.802	0.098	0.025
T_max (obs)_ (h)	4	4	4	4	4
Terminal elimination half-life (h)	3	3.62	3.52	4.88	3.49
λ_z_ (1/h)	0.231	0.192	0.197	0.142	0.198
AUC_0−∞_ (h*μg/mL)	0.436	4.97	4.64	0.644	0.175
AUC extrapolation (%)	10.73	0.462	0.94	8.74	14.69

*C_max (obs)_, indicates maximum observed plasma concentration; T_max (obs)_, observed time to maximum plasma concentration; λ_z_, terminal elimination rate constant; AUC_0−∞_, area under the plasma concentration time curve from time 0 to infinity; MRT_0−∞_, mean residence time. The terminal elimination half-life was calculated based on the best fit data points*.

**Figure 3:**
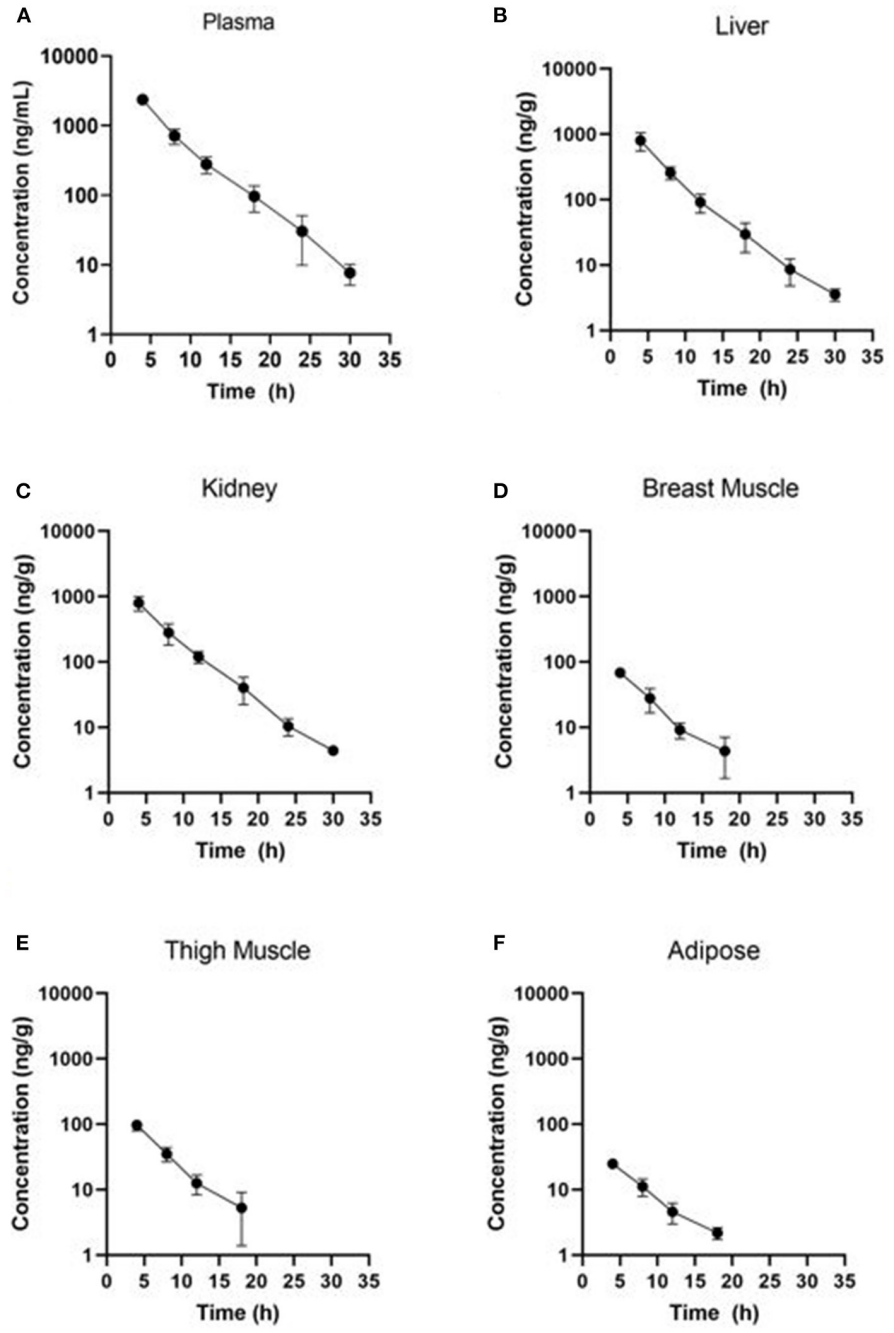
Concentration vs. time curves for tissues collected at slaughter during the IV residue depletion investigation: **(A)** plasma, **(B)** liver, **(C)** kidney, **(D)** breast muscle, **(E)** thigh muscle, and **(F)** adipose. Samples collected from breast muscle, thigh muscle and adipose were below LOD at the 24 and 30 h post-treatment sampling point.

**Figure 4:**
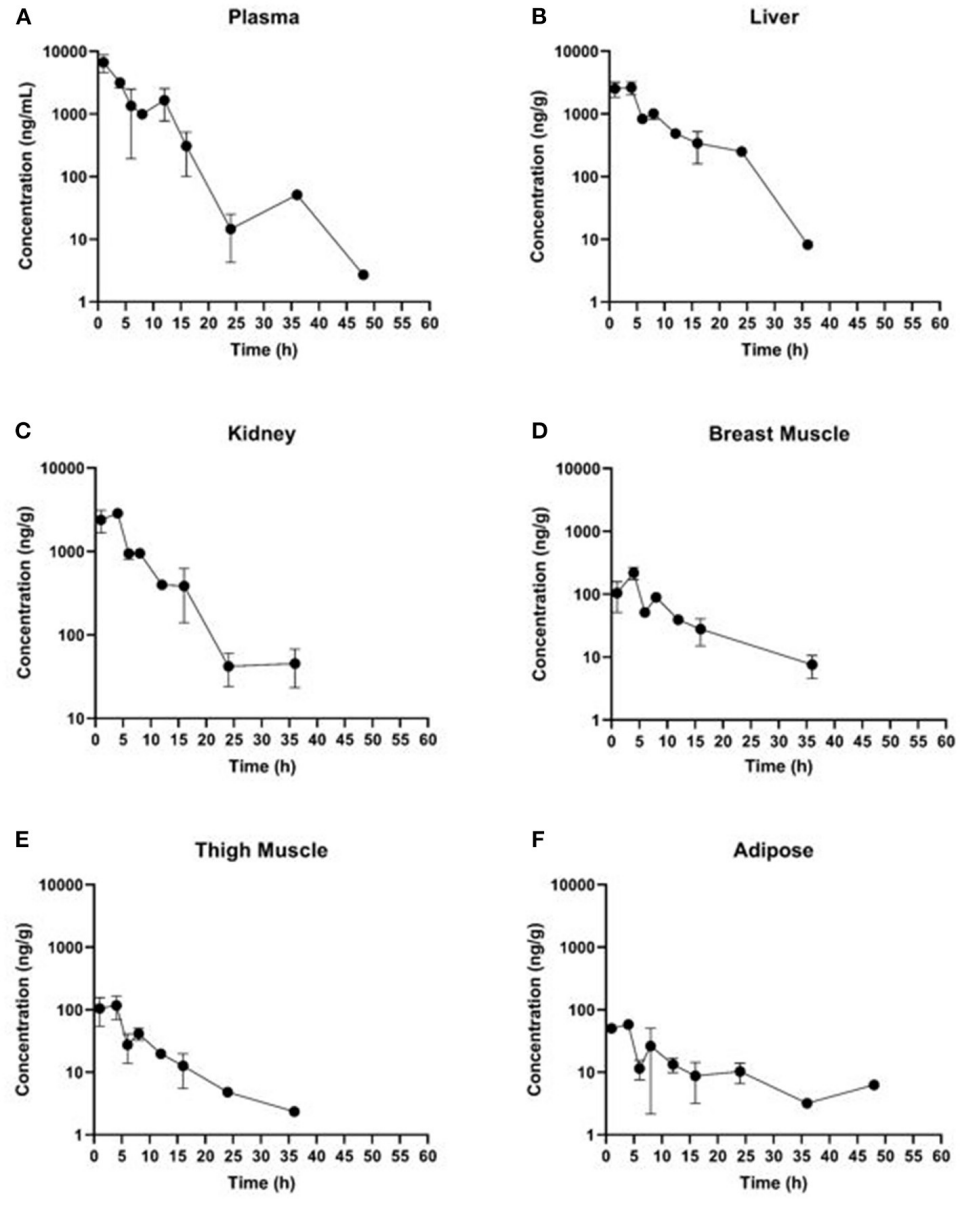
Concentration vs. time curves for tissues collected at slaughter during the PO twice daily dosing regimen group: **(A)** plasma, **(B)** liver, **(C)** kidney, **(D)** breast muscle, **(E)** thigh muscle, and **(F)** adipose.

Estimated ELDU WDIs using the terminal elimination half-life method, as well as the FDA tolerance and EMA MRL methods are presented in Table 5 (IV) and Table 6 (PO). Due to an insufficient number of animals with meloxicam concentrations above LLOQ at a sufficient number of time points, a WDI estimation for some tissue matrices was not possible using either the FDA tolerance or EMA MRL methods (breast and adipose for the IV group, adipose for the PO twice daily dosing regimen group). Figure 3 displays the average concentration vs. time profiles for plasma and tissues sampled at slaughter during the IV portion and Figure 4 displays the average concentration vs. time profiles for plasma and tissues sampled at slaughter during the PO twice daily dosing regimen group. Supplementary Figure 8 displays the average concentration vs. time profiles for plasma and tissues sampled at slaughter during the PO once daily dosing regimen group.

**Table 5:** Estimated preliminary^*^ extra-label drug use (ELDU) withdrawal intervals (WDIs) for meloxicam administered to chickens at 1 mg/kg IV once.

**Method**	**Plasma (live)**	**Plasma (slaughter)**	**Thigh muscle**	**Kidney**	**Liver**	**Breast muscle**	**Adipose**
Terminal elimination half-life method	31 (48)	35 (48)	49 (72)	36 (48)	35 (48)	30^∧^ (48)	35^∧^ (48)
FDA tolerance limit method	37 (48)	39 (48)	22 (24)	37 (48)	31 (48)	NC	NC
EMA maximum residue limit method	30 (48)	28 (48)	15 (24)	24 (24)	22 (24)	NC	NC

*Terminal elimination half-life, Food and Drug Administration Tolerance and European Medicine Agency Maximum Residue Limits methods were used to calculate ELDU WDIs. The calculated ELDU WDI is listed, then rounded up to the nearest 24 h interval to recommend a slaughter time. Plasma and tissue meloxicam concentration over time data are reported in Supplementary Table 3*.

* *ELDU WDI denoted as “preliminary” due to limited number of animals*.

*NC, Not calculated due to lack of sufficient data points with a majority of samples above LLOQ; CI, confidence interval; ^∧^, These matrices only had three time points eligible for calculating the terminal elimination half-life*.

**Table 6:** Estimated extra-label drug use (ELDU) withdrawal intervals (WDIs) for meloxicam administered orally via two dosing regimens in chickens.

**Dosing regimen**	**Method**	**Plasma (slaughter)**	**Thigh muscle**	**Kidney**	**Liver**	**Breast Muscle**	**Adipose**
1 mg/kg PO once daily × 8 doses	Terminal elimination half-life method	35 (48)	33 (48)	34 (48)	43 (48)	42 (48)	82 (96)
	FDA tolerance limit method	57 (72)	49 (72)	46 (48)	58 (72)	36 (48)	78 (96)
	EMA maximum residue limit method	41 (48)	33 (48)	31 (48)	42 (48)	24 (24)	33 (48)
1 mg/kg PO twice a day × 20 doses	Terminal elimination half-life method	47 (48)	47 (48)	60 (72)	45 (48)	103 (120)	373 (384)
	FDA tolerance limit method	34 (48)	42 (48)	73 (96)	62 (72)	77 (96)	NC
	EMA maximum residue limit method	25 (48)	28 (48)	51 (72)	45 (48)	45 (48)	NC

*Terminal elimination half-life, Food and Drug Administration Tolerance and European Medicines Agency Maximum Residue Limits methods were used to calculate ELDU WDIs. The calculated ELDU WDI is listed, then rounded up to the nearest 24 h interval to recommend a slaughter time. Plasma and tissue meloxicam concentration over time data are reported in Supplementary Tables 4–5*.

*NC, Not calculated due to lack of sufficient data points with a majority of samples above LLOQ; CI, confidence interval*.

## Discussion

Meloxicam is commonly used extra-label in urban chickens, despite little scientific data to establish conservative ELDU WDIs. Results from our study indicate that ELDU WDI estimates calculated using three different methods from either plasma or tissue concentration vs. time data are relatively similar and short. For three meloxicam dosing regimens administered to laying hens, the longest ELDU WDIs ranged from 48 to 384 h (or 120 h, if adipose is excluded) using three different WDI estimation methods. The terminal elimination method and adipose tissue resulted in the longest ELDU WDIs. The ELDU WDI for IV administration should be considered as a preliminary estimate due to the limited number of study subjects. This is the first study to use statistical approaches for estimating EDLU WDIs for various routes of meloxicam administration and dosing regimens for laying hens. In addition, our study had added value in showing that depletion curves for plasma and tissue samples following intravenous and oral administrations of meloxicam to laying hens were somewhat similar, thereby indicating that plasma data has the potential to be a starting point for estimating tissue ELDU WDIs. Finally, the data from our study were used to estimate pharmacokinetic parameters for meloxicam administered to laying hens and were similar compared to other bird species.

Generally, using traditional pharmacokinetic methods to estimate the terminal elimination half-life and using this approach to calculate drug depletion resulted in the longest ELDU WDIs for the three treatment regimens when compared to the FDA and EMA statistical methods. Vranic et al. states that when defining a withdrawal period, a safety span could be calculated by multiplying the tissue depletion half-life by 1 to 3 “in order to compensate for the uncertainties of biological variability” (29). However, given that AMDUCA requires a conservative WDI recommendation, we applied a 10-times safety factor with the assumption that >99% of drug is depleted after 10 elimination half-lives (3). This resulted in longer ELDU WDIs for some tissues when using the terminal elimination half-life method compared to those calculated using the regulatory statistical methods. This was especially notable for adipose tissue since meloxicam concentrations for all sampling time points were very close to the LLOQ. In contrast, the regulatory statistical methods have data management guidelines that support eliminating sampling time points with concentrations close to the LLOQ, as long as all of the other criteria for calculating a WDT are fulfilled. For example, the EMA Committee for Medicinal Products for Veterinary Use excluded adipose from meloxicam MRL determinations in bovine species due to the relatively short measurable marker residue concentrations and negligible ratio of residues detected in adipose (30, 31). Similar to bovine species, laying chickens had relatively short measurable meloxicam concentrations in adipose tissue samples therefore ELDU WDIs using the regulatory statistical methods could not be calculated for most dosing regimens.

If a substantial number of animals are treated and the shortest reliable ELDU WDI is necessary to minimize economic loss, then the FDA tolerance and EMA MRL methods offer the advantage of employing confidence intervals to include 99 and 95% of the population, respectively, when calculating a WDT. These regulatory ELDU WDIs theoretically represent ranges of the population mean vs. the sample mean, which is used to calculate the terminal elimination half-life. Furthermore, the ELDU WDIs calculated using these regulatory methods in our study were conservative since we utilized 2-times the LLOQ, given the lack of a chicken MRL. These substituted MRLs were substantially lower than the MRLs established for bovine species. However, if the FDA tolerance or EMA MRL methods are used to estimate ELDU WDIs when large numbers of birds have been treated with a medication in an extra-label manner, the data sets and animal subject numbers should comply with the regulatory guidelines for calculating withdrawal times.

Although our study followed the regulatory FDA guidance regarding data management for statistically calculating WDTs, it is important to note that this study was not intended to estimate a regulatory withdrawal time and therefore did not meet the minimum number of animals for each sampling time point required to establish a regulatory WDT (23, 32). However, the numbers of animals sampled per time point did fulfill the criteria for metabolism studies outlined by the FDA and EMA (23, 33), which is relevant since data from metabolism studies is most commonly used for estimating ELDU WDIs. Furthermore, the minimum required 6 birds per time point for a regulatory tissue residue study is targeting products with an expected zero day withdrawal period which arguably would require more animal subjects to have greater statistical power (32).

Even though meloxicam is not approved by the FDA or EMA for use in avian species, we compared the longest 3, 4, and 16 day ELDU WDIs for IV, PO once daily and PO twice daily, respectively, estimated from our study with those established by the EMA for cattle and swine (34), as well as ELDU WDIs estimated for sheep (24). The EMA established a 15 day meat WDT for cattle following a single 0.5 mg/kg subcutaneous or intravenous dose, and a 5 day meat WDT for swine following 0.4 mg/kg administered intramuscularly or orally up to two times 24 h apart (34). The MRLs established for both cattle and swine are 25 ppb for muscle, and 65 ppb for both liver and kidney. Following an ELDU meloxicam regimen of 1 mg/kg administered orally once a day for ten doses in sheep, Depenbrock et al. estimated a 6 or 10 day meat ELDU WDI using the EMA MRL or FDA tolerance method, respectively (24). Comparatively our ELDU WDIs were similar with the exception for the ELDU WDI for a single 1 mg/kg IV dose administered to laying hens, and this ELDU WDI is recommended as preliminary.

Results from this study suggest that plasma data can be used as a baseline for estimating tissue ELDU WDIs when meloxicam is administered to laying chickens. While plasma data can be used as a proxy for tissue WDIs for meloxicam in chickens, a similar correlation was not seen in eggs, where WDIs were 12 days for egg yolk and 36 days for ovarian follicles (28). The ELDU WDIs estimated from plasma terminal elimination half-lives in the IV live bird and slaughtered bird tissue residue depletion investigations were similar, making the bridge from live bird studies to residue depletion studies. Furthermore, the plasma depletion profiles from slaughtered birds were similar to the live animal studies and did not have differences as was reported in sheep (24). The ELDU WDIs estimated from plasma samples collected at slaughter were similar to other tissues but were never as long as tissues with the slowest depletion for all routes of administration. This finding is consistent with the small volume of distribution noted when meloxicam is administered IV to laying hens, which indicates that meloxicam does not distribute widely outside of the central compartment. However, these findings highlight that ELDU WDIs estimated from published studies where plasma samples were collected will need to add on additional safety factors to account for matrix differences.

Pharmacokinetic parameters calculated in the present study are comparable to those from other published studies in a variety of avian species looking at meloxicam administration by multiple routes in Table 7. Across multiple avian species, the reported plasma terminal elimination half-life for meloxicam varies widely; however, studies using chickens, including this one, report a relatively constant half-life at ~3 h, following IV and oral administration (8, 17–19). In general, this is a longer elimination half-life relative to other avian species that exhibit an approximate 1 h elimination half-life, the main exception being a 5- to 10-fold longer elimination half-life observed in parrots (*Amazona ventralis* and *Psittacus erithacus*) (6, 7). Differences in elimination half-lives may be attributed to differences in metabolism stemming from interspecies variations in cyclooxygenase selectivity, protein binding or biotransformation pathways (37).

**Table 7:** Previously published pharmacokinetic meloxicam studies in various avian species.

**Authors**	**Species**	**Dose and route**	**Terminal half-life (h)**	**Volume of distribution (L/kg)**	**Bioavailability**
Current study	Domestic laying hens	1 mg/kg × 1, IV	3.08	0.19	NR
		1 mg/kg q24h × 8, PO	3.49^*^	0.16^*^	NR
		1 mg/kg q12h × 20, PO	4.74^*^	0.19^*^	NR
Souza et al. (21)	Domestic laying hens (Wyandotte breed)	1 mg/kg × 1, PO	5.53 ± 1.37	NR	NR
Baert and De Backer (18)	Broiler chickens	0.5 mg/kg × 1, IV	3.2	0.12	NR
Souza et al. (8)	Domestic chickens (*Gallus domesticus*)	1 mg/kg × 1, PO	2.79 ± 1.01	NR	NR
Souza et al. (20)	Domestic chickens	1 mg/kg q12h × 9, PO	3.02 ± 1.15	NR	NR
Baert and De Backer (17)	Heavy breed chickens	0.5 mg/kg × 1, IV	3.2	0.058	NR
Baert and De Backer (19)	5 Species	0.5 mg/kg × 1, IV	Pigeon: 2.4; Duck: 0.72; Turkey: 0.99; Ostrich: 0.5; Chicken: 3.21	Pigeon: 0.14 ± 0.1; Duck: 0.065 ± 0.017; Turkey: 0.079 ± 0.015; Ostrich: 0.58 ± 0.19; Chicken: 0.058 ± 0.005	NR
Lacasse et al. (35)	Great horned owls (*Bubo virginianus*) and red-tailed hawks (*Buteo jamaicensis*)	0.5 mg/kg × 1, PO/IV	IV: GHO = 0.78 ± 0.52; RTH = 0.49 ± 0.5; PO: GHO = 5.07 ± 4.5; RTH = 3.97 ± 3.32	IV: GHO = 0.138 ± 0.063; RTH = 0.832 ± 0.711; PO: GHO = 1.150 ± 1.011; RTH = 3.810 ± 5.240	GHO = 62 ± 0.15; RTH = 74 ± 0.48
Molter et al. (6)	Hispaniolan Amazon parrots (*Amazona ventralis*)	1 mg/kg × 1, IV/IM/PO	IV = 15.9 ± 4.4; IM = 15.1 ± 7.7; PO = 15.8 ± 8.6	IV = 0.232 ± 0.22	IM = 100 ± 25; PO = 62 ± 11
Montesinos et al. (7)	African gray parrots (*Psittacus erithacus*)	1 mg/kg × 1, IV/IM/PO	IV = 31.4 ± 4.6; IM = 35.3 ± 6.1; PO = 33.3 ± 3.1	IV = 0.091 ± 0.004	IM = 78.4 ± 5.5; PO = 38.1 ± 3.6
Lindemann et al. (36)	Caribbean flamingos (*Phoenicopterus ruber ruber*)	PO = 3 mg/kg × 1; SC = 1.5 mg/kg × 1	PO = 1.832; SC = 1.104	NR	NR
Boonstra et al. (37)	American flamingos (*Phoenicopterus ruber*)	1 mg/kg × 1, IM/PO	IM = 1.83 ± 1.22; PO = 3.83 ± 2.64	IM = 0.530 ± 0.487; PO = 2.42 ± 1.167	NR
Naidoo et al. (38)	Vultures	2 mg/kg × 1, IM/PO	IM = 0.6 ± 0.15; PO = 0.47 ± 0.25	IM = 0.26; PO = 0.15	NR
Baert et al. (39)	Ostriches (*Struthio camelus*)	0.5 mg/kg × 1, IV	0.54	0.58	NR

*NR, Not reported in the published manuscript*;

* *, pharmacokinetic parameter is determined after the final dose*.

The volume of distribution in the current study is slightly higher than in previous studies completed in chickens (8, 17–19), yet it is more similar to the volume of distribution observed by Molter et al. in Hispaniolan Amazon parrots (6). Despite these variations, overall the volume of distribution of meloxicam following intravenous administration in avian species tends to be low (<1 L/kg). This low volume of distribution can be attributed to high plasma protein binding (~99.4% in humans) and minimal tissue distribution, which is reported for meloxicam in humans and other species (7, 40).

Future meloxicam pharmacokinetic and drug residue elimination studies should be performed in aged backyard hens since differences in pharmacokinetic parameters have been reported for young (< 18 weeks of age) vs. adult birds (41, 42), but geriatric non-laying birds were not evaluated. In addition, efficacy of meloxicam for treating pain should be investigated in a comparable group of chickens to assess the pharmacodynamics of this drug.

## Conclusion

Results from this study indicate that all three methods used to estimate tissue ELDU WDIs resulted in fairly similar results when meloxicam was administered to domestic laying hens. The exception was the longest ELDU WDI of 16 days for the treatment regimen of 1 mg/kg PO twice a day for 20 doses, which was likely an overly conservative WDI given the low meloxicam concentrations in adipose tissue for all sampling points resulting in a longer terminal elimination half-life. Comparison of regulatory statistical methods to estimate tissue ELDU WDIs with ELDU WDIs calculated using terminal elimination half-lives for plasma samples indicates that plasma is suitable as a baseline matrix for estimating edible tissue ELDU WDI for meloxicam in laying chickens. Future meloxicam studies in chickens should focus on multi-dose intravenous and/or oral regimens in clinically painful chickens, as well as establishing methods for estimating withdrawal intervals for eggs. In addition, evaluating whether plasma terminal elimination half-lives reflect drug depletion in eggs would be helpful for estimating egg ELDU WDIs following extra label drug use.

## Data Availability Statement

The original contributions presented in the study are included in the article/Supplementary Material, further inquiries can be directed to the corresponding author/s.

## Ethics Statement

The animal study was reviewed and approved by Institutional Animal Care and Use Committee of the University of California, Davis (protocol number 21269, approved June 27, 2019).

## Author Contributions

LT: conceptualization and project administration. LT, ER, and RD: methodology, data curation, and writing—original draft preparation. ER, SW, and NB: formal analysis. JD, ZL, MC, and SW: writing—review and editing. All authors have read and agreed to the published version of the manuscript.

## Funding

This study was funded by a United Stated Department of Agriculture National Institute of Food and Agriculture grant for the Food Animal Residue Avoidance and Depletion Program.

## Conflict of Interest

RD was employed by Genentech. The remaining authors declare that the research was conducted in the absence of any commercial or financial relationships that could be construed as a potential conflict of interest.

## Publisher's Note

All claims expressed in this article are solely those of the authors and do not necessarily represent those of their affiliated organizations, or those of the publisher, the editors and the reviewers. Any product that may be evaluated in this article, or claim that may be made by its manufacturer, is not guaranteed or endorsed by the publisher.

## Abbreviations

AMDUCA: animal medicinal drug use clarification act
ELDU: extra-label drug use
EMA: european medicines agency
FARAD: food animal residue avoidance databank
FDA: food and drug administration
FSIS: food safety and inspection service
LLOQ: lower limit of quantification
LOD: limit of detection
MRL: maximum residue limit
NSAID: non-steroidal anti-inflammatory drug
TOL: tolerance
WDI: withdrawal interval
WDT: withdrawal time.
