# Supplementary material for: Pharmacokinetic Parameters and Estimating Extra-Label Tissue Withdrawal Intervals Using Three Approaches and Various Matrices for Domestic Laying Chickens Following Meloxicam Administration
Source: Front Vet Sci. 2022 Mar 3;9:826367. doi: 10.3389/fvets.2022.826367 (PMC8927936; doi:10.3389/fvets.2022.826367)
Supplement: Supplementary file 1 [file Data_Sheet_1.docx]

Pharmacokinetic parameters and estimating extra-label tissue withdrawal intervals using three approaches and various matrices for domestic laying chickens following meloxicam administration

Emily D. Richards, PharmD, Rachel S. Dutch, BS, MS, Nathaniel C. Burmas, Jennifer L. Davis, DVM, PhD, Zhoumeng Lin, BMed, PhD, Maaike O. Clapham, BS, Scott E. Wetzlich, BS, Lisa A. Tell, DVM

Supplementary Material

(A)
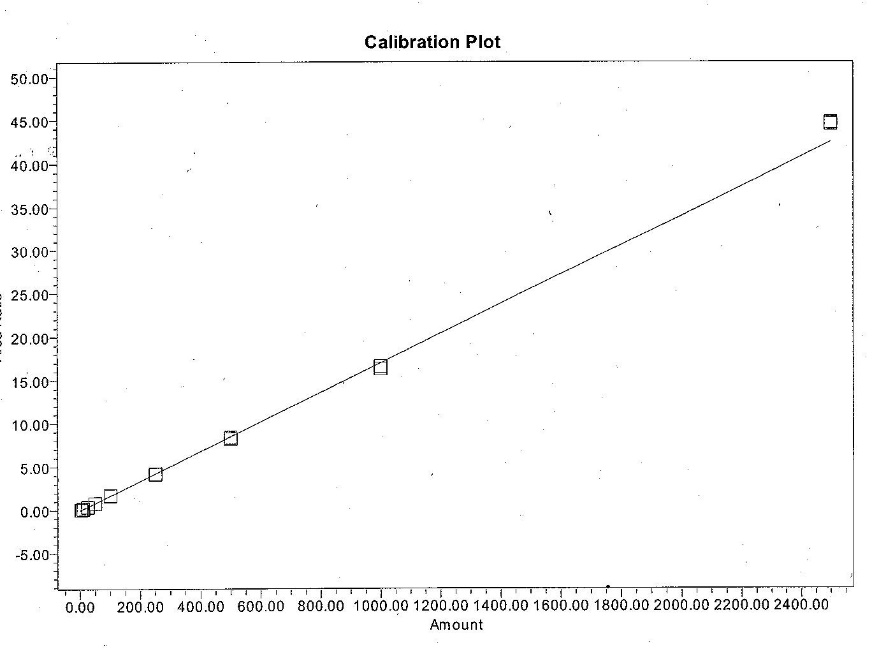


(B)
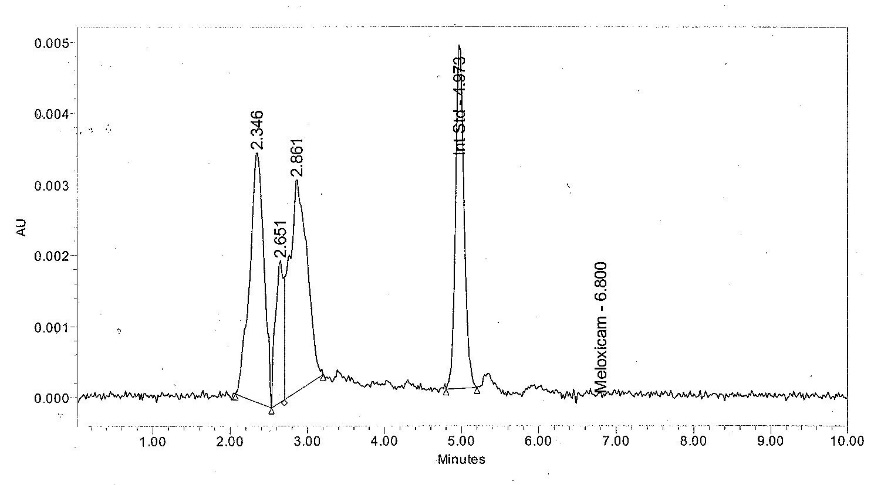


(C)
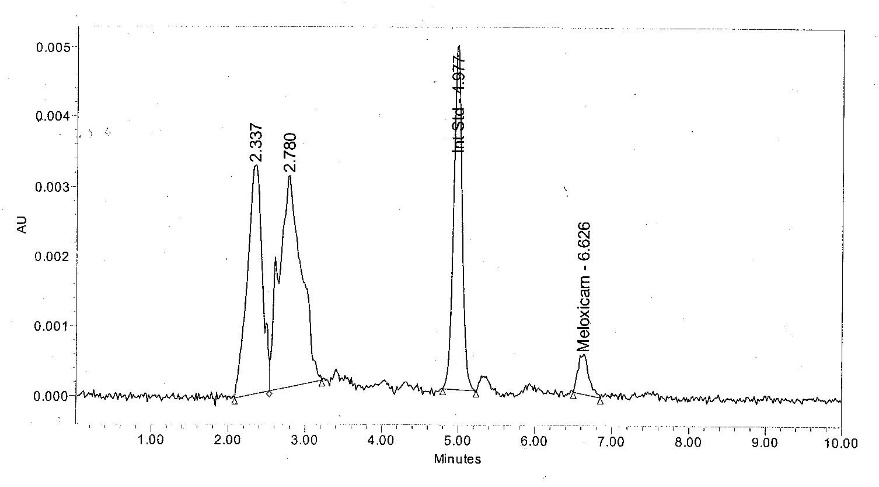


(D)
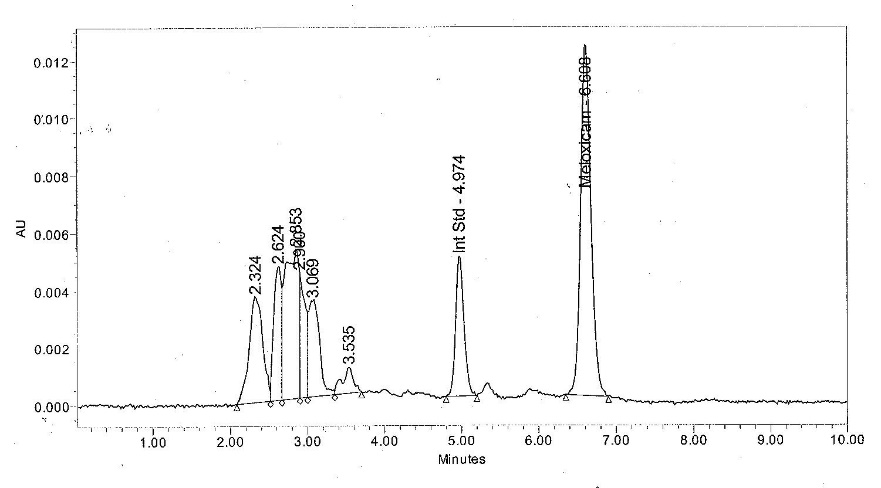


**Supplementary Figure 1**. Representative calibration plot and chromatograms for meloxicam analysis in chicken plasma. **(A)** Representative calibration plot, **(B)** 0 µg/mL quality control sample chromatogram, **(C)** 0.02 µg/mL quality control sample chromatogram, **(D)** incurred sample chromatogram.


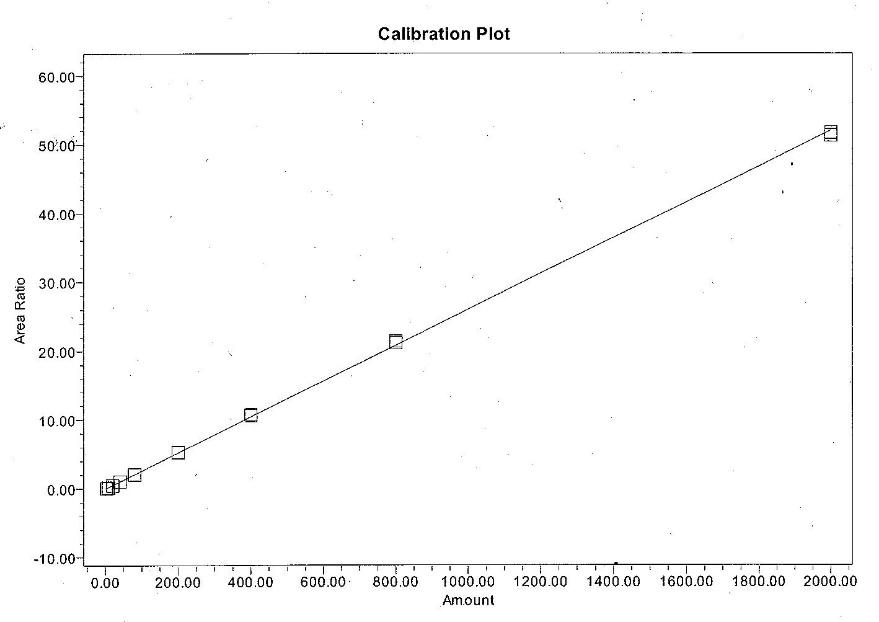


**Supplementary Figure 2**. Representative calibration plot for meloxicam analysis in chicken tissues.

(A)
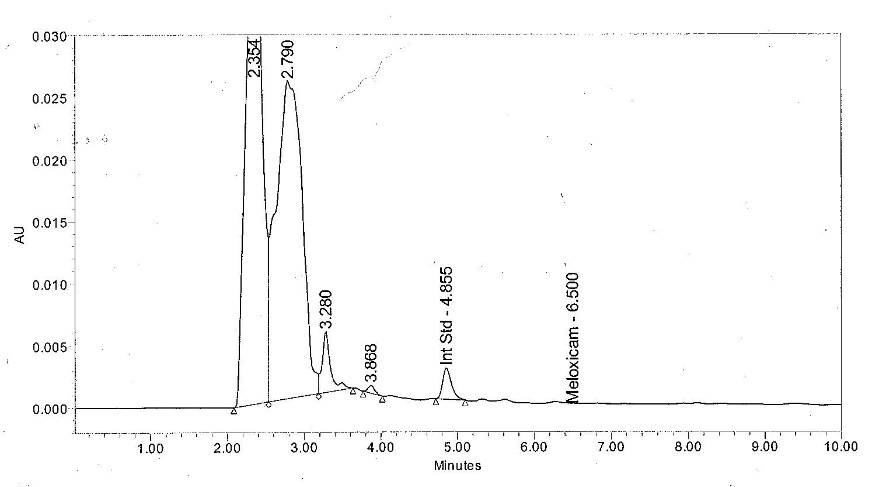


(B)
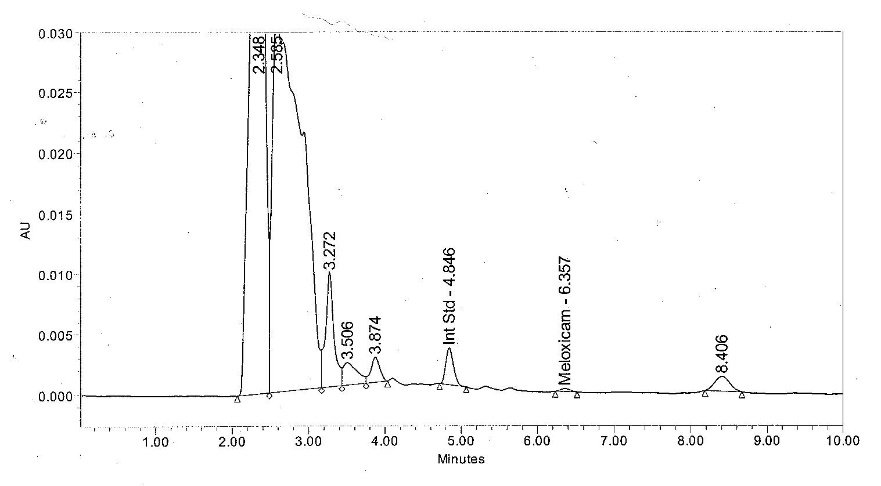


(C)
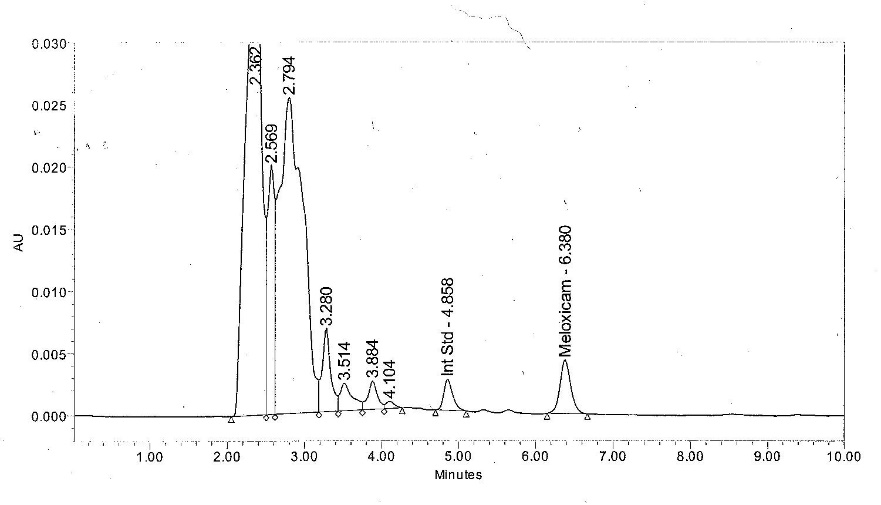


**Supplementary Figure 3**. Representative chromatograms for meloxicam analysis in chicken liver. **(A)** 0 µg/mL quality control sample chromatogram, **(B)** 0.02 µg/mL quality control sample chromatogram, **(C)** incurred sample chromatogram.

(A)
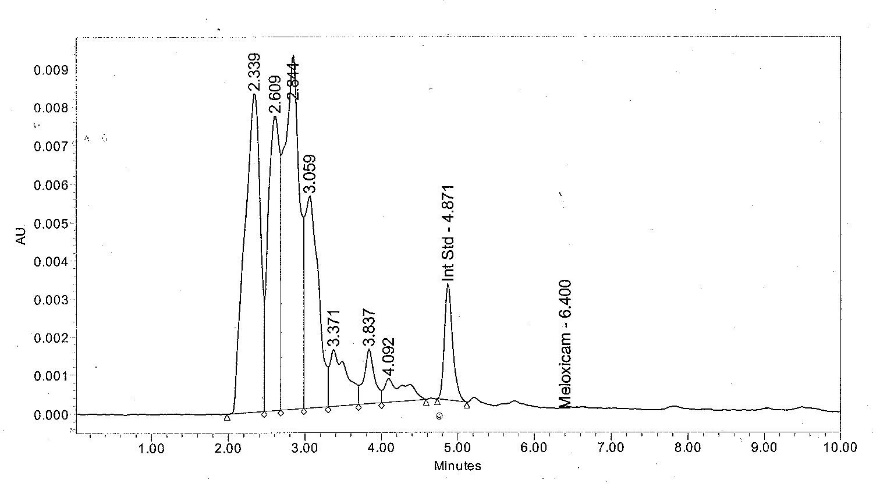


(B)
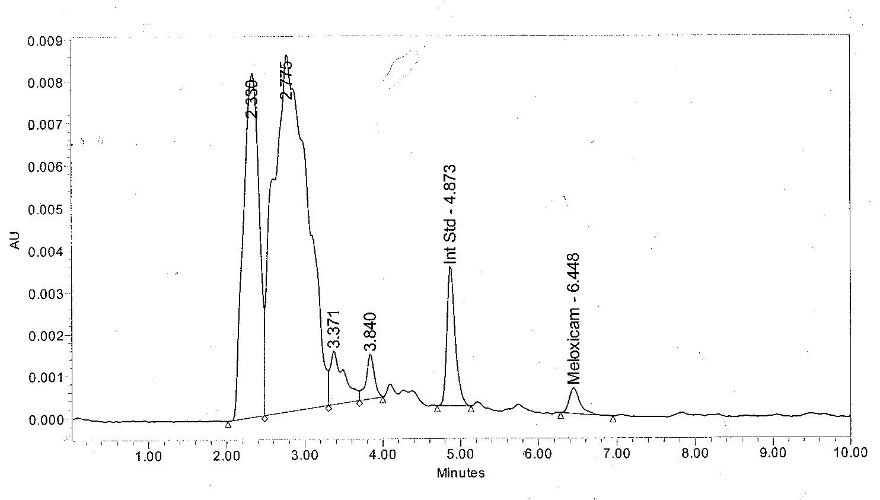


(C)
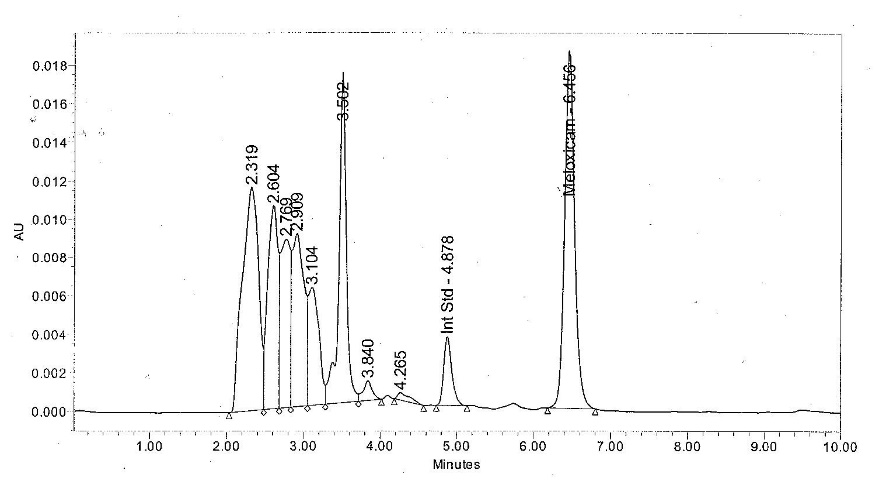


**Supplementary Figure 4**. Representative chromatograms for meloxicam analysis in chicken kidney. **(A)** 0 µg/mL quality control sample chromatogram, **(B)** 0.02 µg/mL quality control sample chromatogram, **(C)** incurred sample chromatogram.

(A)
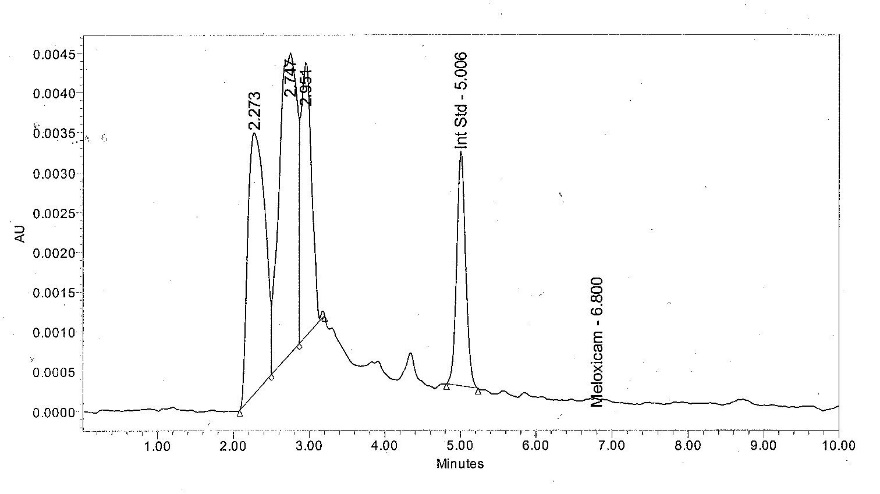


(B)
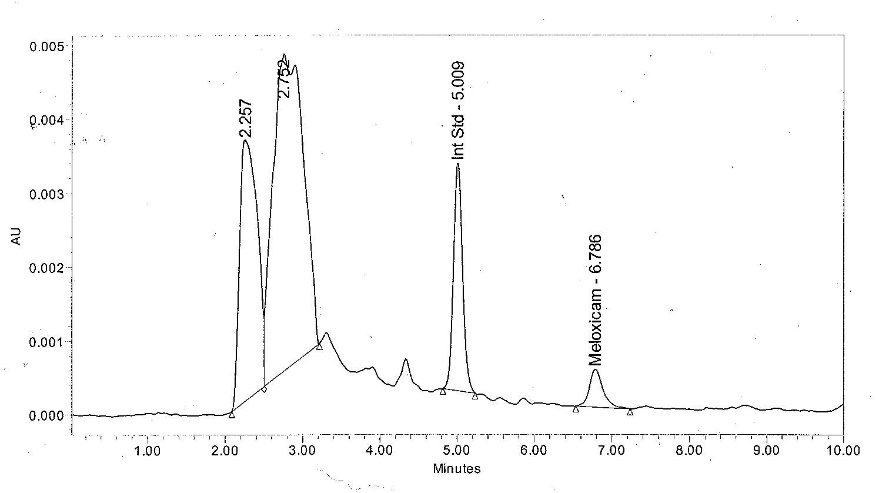


(C)
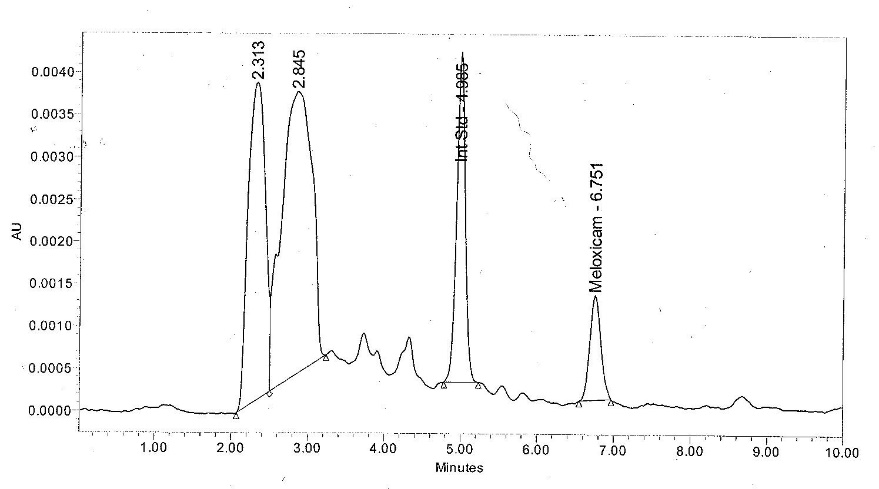


**Supplementary Figure 5**. Representative chromatograms for meloxicam analysis in chicken breast muscle. **(A)** 0 µg/mL quality control sample chromatogram, **(B)** 0.02 µg/mL quality control sample chromatogram, **(C)** incurred sample chromatogram.

(A)
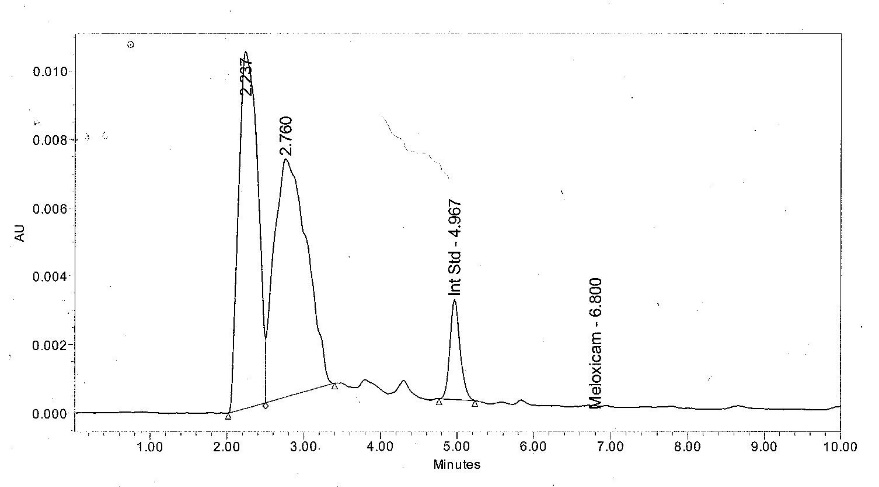


(B)
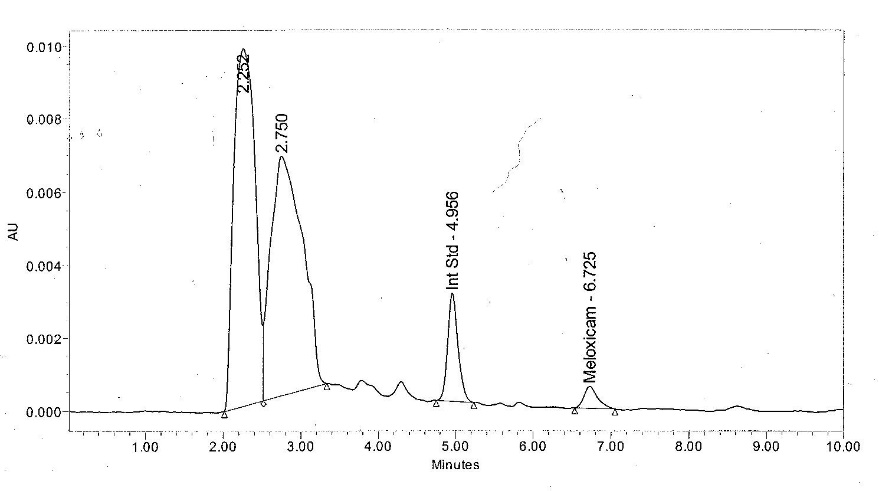


(C)
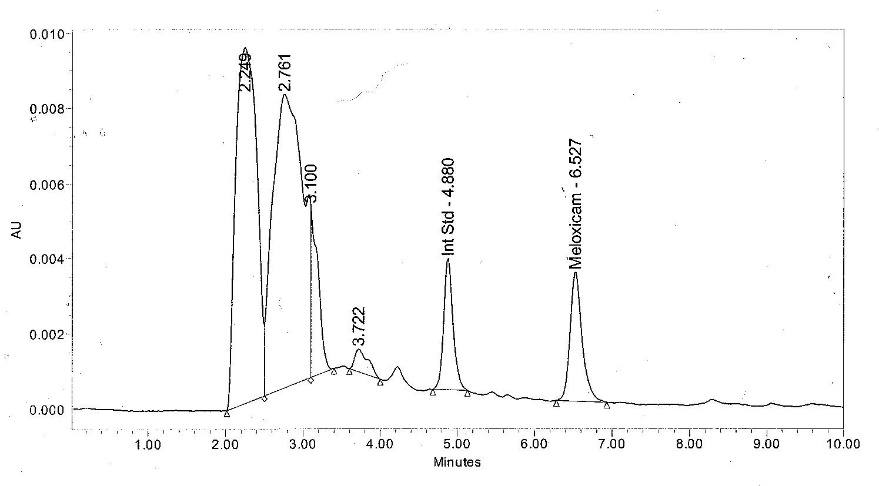


**Supplementary Figure 6**. Representative chromatograms for meloxicam analysis in chicken thigh muscle. **(A)** 0 µg/mL quality control sample chromatogram, **(B)** 0.02 µg/mL quality control sample chromatogram, **(C)** incurred sample chromatogram.

(A)
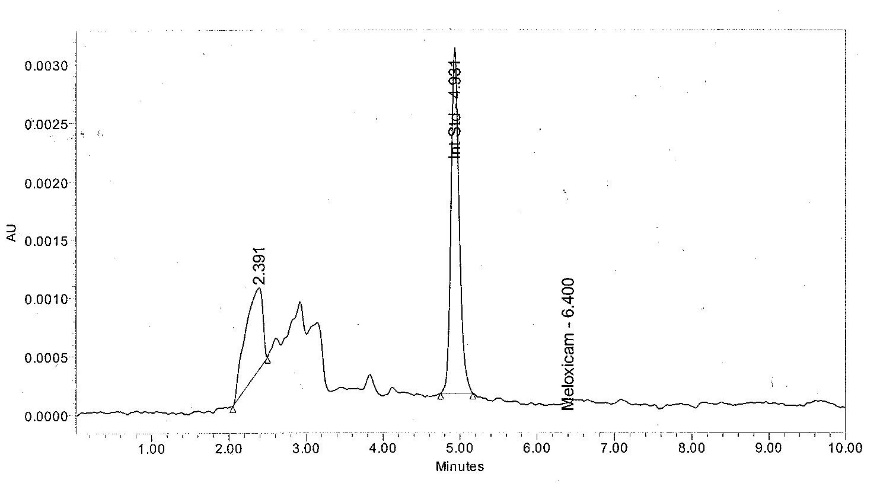


(B)
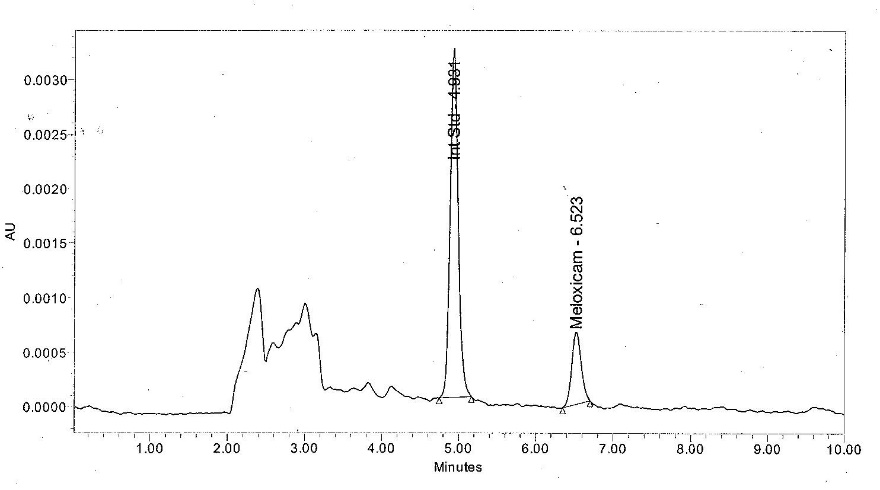


(C)
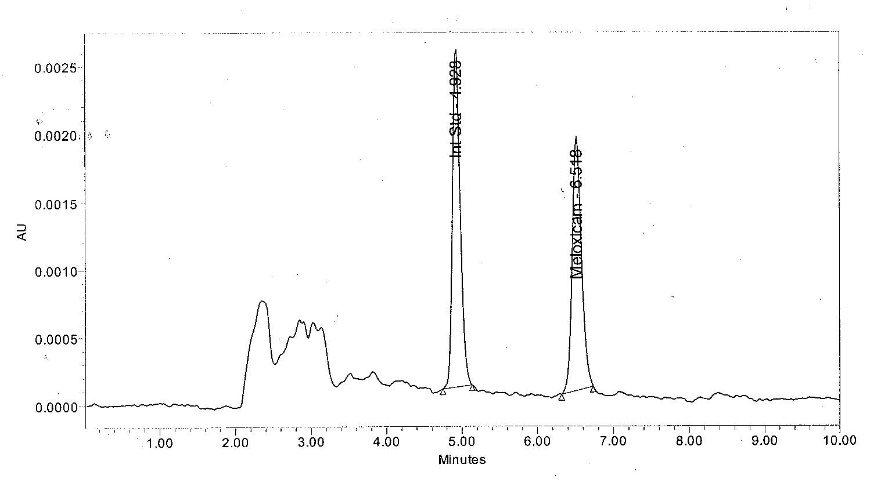


**Supplementary Figure 7**. Representative chromatograms for meloxicam analysis in chicken adipose. **(A)** 0 µg/mL quality control samples chromatogram, **(B)** 0.02 µg/mL quality control sample chromatogram, **(C)** incurred sample chromatogram.

**
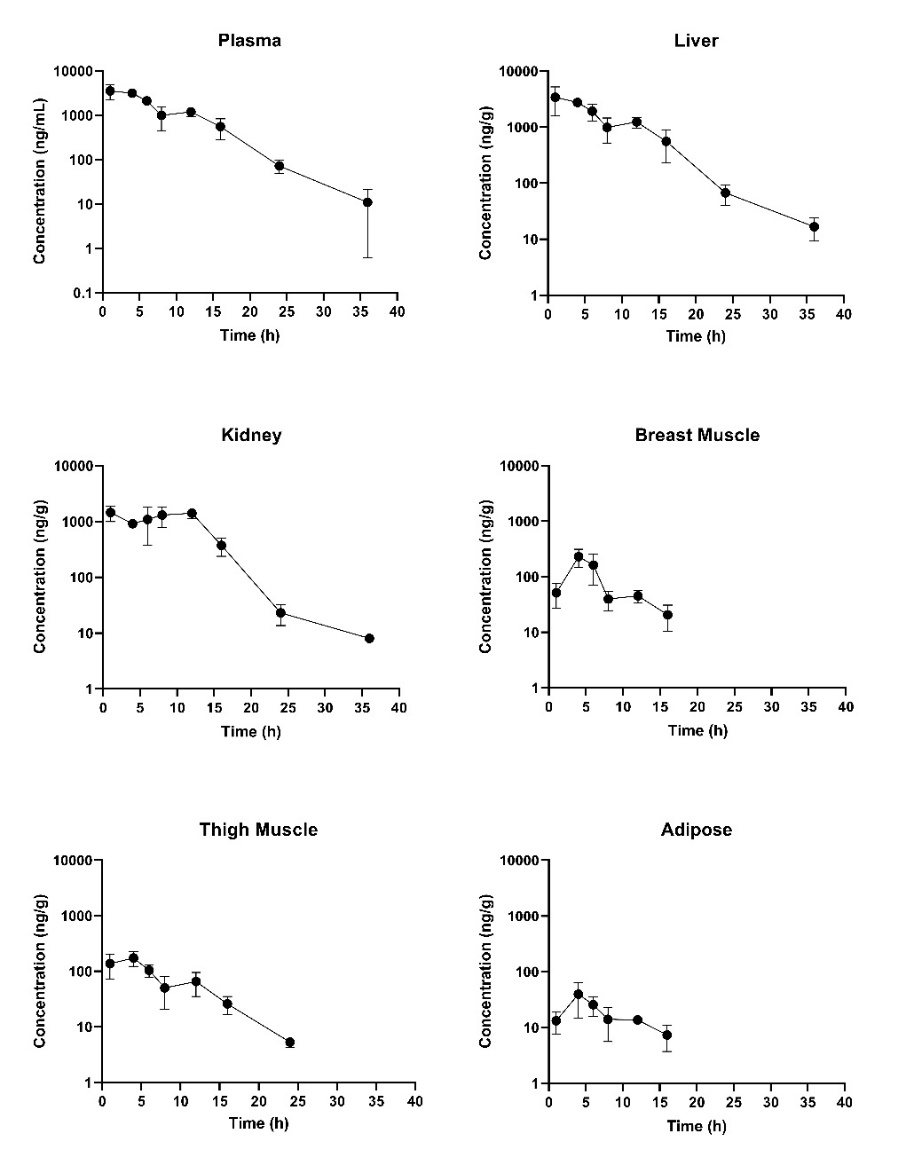
**

**Supplementary Figure 8.** Concentration versus time curves for tissues collected at slaughter during the 1 mg/kg PO once daily investigation in laying hens (from left to right, top to bottom): **(A)** plasma, **(B)** liver, **(C)** kidney, **(D)** breast muscle, **(E)** thigh muscle, **(F)** adipose. Note: samples collected from breast muscle, thigh muscle and adipose were below LOD at the 24 and 30 h post-treatment sampling point.

**Supplementary Table 1.** Organ weights of ten Hy-Line W-36 laying hens, five treated and five untreated.

| **Bird** | **Tx Status** | **BW (kg)** | **Breast Muscle (g)** | | **Thigh Muscle (g)** | | **Kidney (g)** | **Liver (g)** | **Spleen (g)** | **Gizzard (g)** | **Pancreas (g)** | **Lung (g)** | **Gut (g)** | **Adipose (g)** | **Remaining Muscle (g)** | **Uterus (g)** | **Follicles (g)** |
| --- | --- | --- | --- | --- | --- | --- | --- | --- | --- | --- | --- | --- | --- | --- | --- | --- | --- |
|  |  |  | **L** | **R** | **L** | **R** |  |  |  |  |  |  |  |  |  |  |  |
| **1** | **Treated** | 1.8 | 90.5 | 94.2 | 49.9 | 62.5 | 17.3 | 68.96 | 1.5 | 16.5 | 3.2 | 8.3 | 48.8 | NC | NC | NC | NC |
| **2** |  | 1.6 | 94.9 | 90.2 | 53.8 | 55.9 | 15.4 | 53.1 | 1.5 | 14.0 | 3.1 | 12.3 | 23.96 | NC | NC | NC | NC |
| **3** |  | 1.7 | 93. 9 | 89.96 | 40.1 | 44.4 | 14.4 | 59.6 | 2.0 | 15.5 | 3.3 | 7.3 | 32.1 | NC | NC | NC | NC |
| **4** |  | 1.8 | 96.3 | 91.9 | 59.1 | 59.4 | 15.7 | 49.6 | 1.2 | 13.8 | 2.6 | 11.2 | 41.6 | NC | NC | NC | NC |
| **5** |  | 1.8 | 105.8 | 104.8 | 66.3 | 66.8 | 20.95 | 73.7 | 1.9 | 16.8 | 4.4 | 12.9 | 35.0 | 272.8 | 183.7 | 76.6 | 71.2 |
| **6** | **Non-treated** | 1.6 | 86.7 | 83.2 | 51.5 | 56.96 | 17.4 | 59.4 | 1.2 | 16.97 | 3.8 | 9.98 | 28.1 | 215.8 | 162.9 | 72.6 | 58.1 |
| **7** |  | 2.1 | 106.8 | 105.98 | 66.1 | 71.1 | 19.2 | 66.6 | 1.7 | 19.3 | 4.1 | 12.2 | 36.2 | 414.8 | 192.1 | 68.3 | 63.4 |
| **8** |  | 2.1 | 112.5 | 115.1 | 71.8 | 71.1 | 20.7 | 81.0 | 2.6 | 16.7 | 5.1 | 11 | 35.2 | 413.3 | 181.5 | 75.3 | 53.2 |
| **9** |  | 2 | 114.4 | 106.8 | 73 | 72.1 | 24.3 | 81.3 | 2.8 | 16.4 | 5.2 | 11.1 | 49.7 | 273.1 | 180.99 | 75.0 | 77.9 |
| **10** |  | 1.6 | 86.96 | 81.7 | 52.7 | 50.4 | 15.4 | 53.7 | 1.3 | 17.2 | 3.5 | 9.7 | 30.1 | 210.5 | 156.2 | 76.4 | 51.8 |
| **Average** | | 1.8 | 98.9 | 96.4 | 58.5 | 61.1 | 18.1 | 64.7 | 1.8 | 16.3 | 3.8 | 10.6 | 36.0 | 300.0 | 176.2 | 74.0 | 62.6 |
| **Standard Deviation** | | 0.2 | 9.7 | 10.6 | 10.2 | 8.9 | 3.0 | 10.9 | 0.5 | 1.5 | 0.8 | 1.7 | 8.0 | 84.3 | 12.5 | 2.9 | 9.4 |

Tx = treatment; BW = body weight; NC = tissues not collected; L = left; R = right.

**Supplementary Table 2.** Meloxicam concentrations measured in plasma samples collected from live laying hens and from laying hens slaughtered during the tissue depletion study phase (1mg/kg IV once). The preliminary (due to limited numbers of birds) ELDU WDI estimate represents the mean plasma elimination half-life multiplied by 10 to represent elimination of >99% of drug.^1^

|  | **Meloxicam Plasma Concentrations (live birds)** | | | | **Meloxicam Plasma Concentrations (at slaughter)** | | | |
| --- | --- | --- | --- | --- | --- | --- | --- | --- |
| **Time (h)** | **Bird 1** | **Bird 2** | **Bird 3** | **Bird 4** | **Bird 1** | **Bird 2** | **Bird 3** | **Bird 4** |
| 0.083 | 8672.8 | 8802.5 | 7130.3 | 7778.2 | * | * | * | * |
| 0.25 | 6431.8 | 7223 | 6327.6 | 6397.3 | * | * | * | * |
| 0.5 | 5897.4 | 6113.8 | 4906.7 | 4896.9 | * | * | * | * |
| 1 | 4663.6 | 4999.2 | 4165.4 | 3418.1 | * | * | * | * |
| 2 | 3485.1 | 3911 | 2966.2 | 2694.8 | * | * | * | * |
| 3 | 2941.7 | 3342.6 | 2493.4 | † | * | * | * | * |
| 4 | 2243.9 | 2658.9 | 1632.4 | 1369.4 | 2775.3 | 2062.5 | 2475.4 | 2143.6 |
| 6 | 1648.3 | 1701.2 | 995.6 | 797.3 | * | * | * | * |
| 8 | 1041.3 | 1013.5 | 574.7 | 401.4 | 501.6 | 649.7 | 781.9 | 914.4 |
| 12 | 401.4 | 411.9 | 248.3 | 133.8 | 266.6 | 385.7 | 253.1 | 209.5 |
| 18 | 130.8 | 88.5 | 53.5 | 31.6 | 93.6 | 49.6 | 97.6 | 146.7 |
| 24 | 60.2 | 27.2 | 19.7 | 14.6 | * | * | * | * |
| 30 | * | * | * | * | 7 | 4.4 | 10.3 | 9 |
| Overall Preliminary ELDU WDI estimate (WDI rounded up to nearest 24 h interval) | 30.8 (48) | | | | 34.5 (48) | | | |

* = sample not collected at associated time point due to study design; † = sample not recorded.

^1^ Riviere, J. E., A. I. Webb and A. L. Craigmill (1998). "Primer on estimating withdrawal times after extralabel drug use." J Am Vet Med Assoc **213**(7): 966-968.

**Supplementary Table 3.** Mean plasma and tissue meloxicam concentrations (ng/mL) over time for meloxicam in chickens (1 mg/kg IV). The number of animals with quantifiable data based on the FDA^1^ or EMA^2^ methods are represented in parentheses for each data time point.

| **Method** | **Criteria** | **Time**  **(h)** | **Plasma (live)** | **Plasma (slaughter)** | **Thigh Muscle** | **Kidney** | **Liver** | **Breast Muscle** | **Adipose** |
| --- | --- | --- | --- | --- | --- | --- | --- | --- | --- |
| Terminal Elimination Half-Life Method | Naïve-pooled terminal elimination half-life multiplied by 10. |  | 31.1 | 34.5 | 48.8 | 36.2 | 35.2 | 27.6^ | 34.9^ |
| FDA Tolerance Limit Method | 95% CI that 99% of animals are included. Limit of detection (LOD) was used in place of a tolerance. | 4 | 1976.2 (4) | 2364.2 (4) | 97.6 (4) | 797 (4) | 802 (4) | 68.4 (4) | 24.9 (4) |
|  |  | 8 | 757.7 (4) | 711.9 (4) | 35.1 (4) | 281.3 (4) | 258.4 (4) | 27.9 (4) | 11.2 (4) |
|  |  | 12 | 298.9 (4) | 278.7 (4) | 12.5 (4) | 119.6 (4) | 91.4 (4) | † | † |
|  |  | 18 | 76.1 (4) | 96.9 (4) | * | 40.2 (4) | 29.7 (4) | † | § |
|  |  | 24 | 30.4 (4) | 30.4 (4) | § | † | † | § | § |
|  |  | 30 | Not collected as part of the study design | † | § | † | § | § | § |
| EMA Maximum Residue Limit Method | 95% CI that 95% of animals are included. Concentrations less than the lower limit of quantification (LLOQ) were substituted with a value of LLOQ/2. LLOQ*2 was used in place of the maximum residue limit. | 4 | 1976.2 (4) | 2364.2 (4) | 97.6 (4) | 797 (4) | 802 (4) | 68.4 (4) | 24.9 (4) |
|  |  | 8 | 757.7 (4) | 711.9 (4) | 35.1 (4) | 281.3 (4) | 258.4 (4) | 27.9 (4) | 9.4 (4) |
|  |  | 12 | 298.9 (4) | 278.7 (4) | 12.5 (4) | 119.6 (4) | 91.4 (4) | † | † |
|  |  | 18 | 76.1 (4) | 96.9 (4) | * | 40.2 (4) | 29.7 (4) | † | § |
|  |  | 24 | 30.4 (4) | 28.7 (4) | § | † | † | § | § |
|  |  | 30 | Not collected as part of the study design | † | § | † | § | § | § |

CI = confidence interval; ^ = calculated using all 3 remaining time points, rather than best fit; * = time point dropped from WDI calculation since excluded from best fit; † = time point dropped from WDI calculation because a majority of samples less than LLOQ; § = time point dropped from WDI calculation because all samples below LOD.

^1^ CVM Guidance for Industry #3: General Principles for Evaluating the Human Food Safety of New Animal Drugs Used in Food-Producing Animals. 2018. Rockville, Maryland: F.D.A.

^2^ Guideline on determination of withdrawal periods for edible tissues. 2018. London, United Kingdom: European Medicines Agency.

**Supplementary Table 4.** Mean plasma and tissue meloxicam concentrations (ng/mL) over time for meloxicam in laying hens (1 mg/kg PO once daily for 8 doses). The number of animals with quantifiable data based on the FDA^1^ or EMA^2^ methods are represented in parentheses for each data time point. Estimated withdrawal intervals were calculated for each matrix are presented using the different methods. For all methods, concentrations less than the limit of detection (LOD) were not used.

| **Method** | **Criteria** | **Time**  **(h)** | **Plasma (slaughter)** | **Thigh Muscle** | **Kidney** | **Liver** | **Breast Muscle** | **Adipose** |
| --- | --- | --- | --- | --- | --- | --- | --- | --- |
| Terminal Elimination Half-Life Method | Naïve-pooled terminal elimination half-life multiplied by 10. |  | 34.9 | 33.3 | 33.6 | 42.5 | 42.05 | 81.7 |
| FDA Tolerance Limit Method | 95% CI that 99% of animals are included. Limit of detection (LOD) was used in place of a tolerance. | 1 | 3541.2 (3) | * | 1443.6 (3) | 3414.4 (3) | * | * |
|  |  | 4 | 3123.4 (3) | 172.9 (3) | 912 (3) | 2764 (3) | 231.2 (3) | 39.7 (3) |
|  |  | 6 | 2111.1 (3) | 104.4 (3) | 1091.3 (3) | 1924.4 (3) | 161.8 (3) | 25.8 (3) |
|  |  | 8 | 993.9 (3) | * | * | 992.7 (3) | * | * |
|  |  | 12 | 1190.8 (3) | 65.3 (3) | * | 1239.7 (3) | 45.4 (3) | 13.7 (3) |
|  |  | 16 | 552.4 (3) | 25.9 (3) | * | 558.7 (3) | 20.6 (3) | ‡ |
|  |  | 24 | 72.0 (3) | ‡ | 23.1 (3) | 67.4 (3) | § | § |
|  |  | 36 | ‡ | § | § | ‡ | § | § |
| EMA Tolerance Limit Method | 95% CI that 95% of animals are included. Concentrations less than the lower limit of quantification (LLOQ) were substituted with a value of LLOQ/2. LLOQ*2 was used in place of the maximum residue limit. | 1 | 3541.2 (3) | * | 1443.6 (3) | † | * | * |
|  |  | 4 | 3123.4 (3) | 172.9 (3) | 912.0 (3) | 2764.0 (3) | 231.2 (3) | 39.7 (3) |
|  |  | 6 | 2111.1 (3) | 104.4 (3) | 1091.3 (3) | 1924.4 (3) | 161.8 (3) | 25.8 (3) |
|  |  | 8 | 993.9 (3) | * | * | 992.7 (3) | * | * |
|  |  | 12 | 1190.8 (3) | 65.3 (3) | * | 1239.7 (3) | 45.4 (3) | 13.7 (3) |
|  |  | 16 | 552.4 (3) | 25.9 (3) | * | 558.7 (3) | 20.6 (3) | ‡ |
|  |  | 24 | 72.0 (3) | ‡ | 21.4 (3) | 67.4 (3) | § | § |
|  |  | 36 | ‡ | § | § | ‡ | § | § |

CI = confidence interval; § = time point dropped from WDI calculation because sample was below LOD; * = time point dropped due to not conforming to linearity; † = time point dropped due to software limitations (maximum 7 time points allowed for analysis); ‡ = time point dropped from WDI calculation because a majority of data points were below LLOQ.

^1^ CVM Guidance for Industry #3: General Principles for Evaluating the Human Food Safety of New Animal Drugs Used in Food-Producing Animals. 2018. Rockville, Maryland: F.D.A.

^2^ Guideline on determination of withdrawal periods for edible tissues. 2018. London, United Kingdom: European Medicines Agency.

**Supplementary Table 5.** Mean plasma and tissue meloxicam concentrations (ng/mL) over time for meloxicam in chickens (1 mg/kg PO twice daily for 20 doses). The number of animals with quantifiable data based on the FDA^1^ or EMA^2^ methods are represented in parentheses for each data time point. Estimated withdrawal intervals were calculated for each tissue are presented using the different methods. For all methods, concentrations less than the limit of detection (LOD) were not used.

| **Method** | **Criteria** | **Time**  **(h)** | **Plasma (slaughter)** | **Thigh Muscle** | **Kidney** | **Liver** | **Breast Muscle** | **Adipose** |
| --- | --- | --- | --- | --- | --- | --- | --- | --- |
| Terminal Elimination Half-Life Method | Naïve-pooled terminal elimination half-life multiplied by 10. |  | 47.4 | 46.8 | 59.5 | 44.8 | 103.2 | 372.5 |
| FDA Tolerance Limit Method | 95% CI that 99% of animals are included. Limit of detection (LOD) was used in place of a tolerance. | 1 | 6692.3 (3) | 105.3 (3) | 2391.2 (3) | 2528.4 (3) | 104.5 (3) | 50.5 (3) |
|  |  | 4 | 3170.5 (3) | 117.6 (3) | 2859.6 (3) | 2621.5 (3) | 219 (3) | 58.1 (3) |
|  |  | 6 | * | * | 945 (3) | 832.5 (3) | 51.4 (3) | * |
|  |  | 8 | 995.6 (3) | 41.5 (3) | 949 (3) | 1014.9 (3) | 88.9 (3) | * |
|  |  | 12 | * | 19.7 (2) | 399.1 (2) | 486.6 (2) | 39.3 (2) | * |
|  |  | 16 | * | 12.7 (3) | 384.9 (3) | 342.5 (3) | 27.8 (3) | * |
|  |  | 24 | 20.6 (2) | § | 42.1 (2) | § | § | * |
|  |  | 36 | * | § | 45.5 (3) | § | ‡ | § |
|  |  | 48 | § | § | § | § | § | ‡ |
| EMA Tolerance Limit Method | 95% CI that 95% of animals are included. Concentrations less than the lower limit of quantification (LLOQ) were substituted with a value of LLOQ/2. LLOQ*2 was used in place of the maximum residue limit. | 1 | 6692.3 (3) | 105.3 (3) | † | 2528.4 (3) | 104.5 (3) | 50.5 (3) |
|  |  | 4 | 3170.5 (3) | 117.6 (3) | 2859.6 (3) | 2621.5 (3) | 219 (3) | 58.1 (3) |
|  |  | 6 | * | * | 945 (3) | 832.5 (3) | 51.4 (3) | * |
|  |  | 8 | 995.6 (3) | 41.5 (3) | 949 (3) | 1014.9 (3) | 88.9 (3) | * |
|  |  | 12 | * | 19.7 (2) | 399.1 (2) | 486.6 (2) | 39.3 (2) | * |
|  |  | 16 | * | 12.2 (3) | 384.9 (3) | 342.5 (3) | 27.8 (3) | *‡ |
|  |  | 24 | 20.6 (2) | § | 42.1 (2) | § | § | *‡ |
|  |  | 36 | * | § | 45.5 (3) | § | ‡ | § |
|  |  | 48 | § | § | § | § | § | ‡ |

CI = confidence interval; § = time point dropped from WDI calculation because a majority of samples were below LOD; * = time point dropped due to not conforming to linearity; † = time point dropped due to software limitations (maximum 7 time points allowed for analysis); ‡ = time point dropped from WDI calculation because a majority of data points were below LLOQ.

^1^ CVM Guidance for Industry #3: General Principles for Evaluating the Human Food Safety of New Animal Drugs Used in Food-Producing Animals. 2018. Rockville, Maryland: F.D.A.

^2^ Guideline on determination of withdrawal periods for edible tissues. 2018. London, United Kingdom: European Medicines Agency.
